# The Zinc Transporter SLC39A10 Plays an Essential Role in Embryonic Hematopoiesis

**DOI:** 10.1002/advs.202205345

**Published:** 2023-04-17

**Authors:** Xuyan He, Chaodong Ge, Jun Xia, Zhidan Xia, Lu Zhao, Sicong Huang, Rong Wang, Jianwei Pan, Tao Cheng, Peng‐Fei Xu, Fudi Wang, Junxia Min

**Affiliations:** ^1^ The First Affiliated Hospital The Second Affiliated Hospital Institute of Translational Medicine School of Public Health State Key Laboratory of Experimental Hematology Zhejiang University School of Medicine 310058 Hangzhou China; ^2^ The First Affiliated Hospital Basic Medical Sciences School of Public Health Hengyang Medical School University of South China 421001 Hengyang China; ^3^ State Key Laboratory of Membrane Biology,Institute of Zoology, Institute for Stem Cell and Regeneration Chinese Academy of Sciences, University of Chinese Academy of Sciences 100101 Beijing China; ^4^ Women's Hospital, and Institute of Genetics Zhejiang University School of Medicine Hangzhou Zhejiang 310058 China

**Keywords:** anemia, hematopoiesis, hematopoietic stem cells, HSPCs, SLC39A10, zinc homeostasis, ZIP10

## Abstract

The role of zinc in hematopoiesis is currently unclear. Here, SLC39A10 (ZIP10) is identified as a key zinc transporter in hematopoiesis. The results show that in zebrafish, *Slc39a10* is a key regulator of the response to zinc deficiency. Surprisingly, both *slc39a10* mutant zebrafish and hematopoietic *Slc39a10*‐deficient mice develop a more severe form of impaired hematopoiesis than animals lacking transferrin receptor 1, a well‐characterized iron gatekeeper, indicating that zinc plays a larger role than iron in hematopoiesis, at least in early hematopoietic stem cells (HSCs). Furthermore, it is shown that loss of *Slc39a10* causes zinc deficiency in fetal HSCs, which in turn leads to DNA damage, apoptosis, and G_1_ cell cycle arrest. Notably, zinc supplementation largely restores colony formation in HSCs derived from hematopoietic *Slc39a10*‐deficient mice. In addition, inhibiting necroptosis partially restores hematopoiesis in mouse HSCs, providing mechanistic insights into the requirement for zinc in mediating hematopoiesis. Together, these findings indicate that SLC39A10 safeguards hematopoiesis by protecting against zinc deficiency‐induced necroptosis, thus providing compelling evidence that SLC39A10 and zinc homeostasis promote the development of fetal HSCs. Moreover, these results suggest that SLC39A10 may serve as a novel therapeutic target for treating anemia and zinc deficiency‐related disorders.

## Graphical abstract

*Slc39a10* (Zip10), as a zinc transporter, plays an essential role in the survival of fetal hematopoietic stem/progenitor cells in both zebrafish and mice. Loss of *Slc39a10* causes impaired hematopoiesis that can be rescued by zinc supplementation or inhibiting necroptosis. This study shows that *Slc39a10* promotes the development of fetal hematopoietic stem cells by maintaining zinc homeostasis.

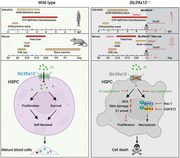

## 1 Introduction

Hematopoiesis is a highly dynamic process that includes the proliferation, differentiation, and maturation of hematopoietic stem cells (HSCs), processes that are tightly controlled by a variety of factors.^[^ ^1^, ^2^, ^3^ ^]^ The development of the mammalian hematopoietic system is complex and—in many respects—poorly understood. In vertebrates, embryonic hematopoiesis can be separated into two stages known as primitive hematopoiesis and definitive hematopoiesis.^[^ ^1^, ^4^ ^]^ The earliest population of definitive HSCs is derived from the ventral wall of the dorsal aorta in the aorta‐gonad‐mesonephros (AGM) region via a process known as the endothelial‐to‐hematopoietic transition.^[^ ^5^, ^6^, ^7^ ^]^ Definitive HSCs subsequently migrate into a transient fetal hematopoietic region for rapid expansion and differentiation; in mammals and zebrafish, this process occurs in the fetal liver (FL) and caudal hematopoietic tissue (CHT), respectively.^[^ ^8^, ^9^, ^10^, ^11^ ^]^ Finally, the HSCs are released into the circulating blood and home to the bone marrow and kidneys in mammals and zebrafish, respectively. Given that fetal HSCs are extremely rare, reliable, sensitive markers expressed exclusively on these cells—particularly in the early developmental stage—have not been identified, thus hindering our ability to functionally study the developmental processes that occur during hematopoiesis.^[^ ^12^ ^]^ We, therefore, used two robust models, zebrafish and mice, to study the formation of definitive HSCs.

Zinc homeostasis is essential for a wide range of physiological functions in nearly all tissues. According to the World Health Organization, zinc deficiency is one of the most prevalent nutritional disorders, with approximately one‐third of the world's population currently affected by zinc deficiency.^[^ ^13^ ^]^ Intracellular zinc homeostasis is regulated primarily by two families of metal transporter proteins, namely the solute‐linked carrier 30 (SLC30A, also known as ZnT) family and the solute‐linked carrier 39 (SLC39A, also known as the Zrt‐ and Irt‐like proteins, or ZIP) family. SLC39A proteins regulate the influx of zinc into the cytoplasm, while SLC30A proteins primarily regulate the efflux of cytoplasmic zinc.^[^ ^14^, ^15^ ^]^ SLC39A10 (also known as ZIP10) was first identified as a putative zinc importer in the brush‐border membrane in rat kidneys, and its expression was shown to be regulated by zinc.^[^ ^16^ ^]^ In addition, we previously found that SLC39A10 is essential for promoting the survival of macrophages via a zinc/p53‐dependent axis following stimulation with lipopolysaccharide (LPS).^[^ ^17^ ^]^ Together, these findings suggest that SLC39A10 plays a key role in regulating zinc homeostasis; however, whether SLC39A10 plays a functional role in hematopoiesis is currently unknown.

The integrity of the immune system has been tightly linked to zinc status, and brief periods of zinc supplementation have been shown to substantially improve the immune response.^[^ ^18^, ^19^ ^]^ Approximately 6 decades ago, Prasad and colleagues first reported that zinc deficiency is associated with anemia,^[^ ^20^, ^21^ ^]^ and since this early report, numerous studies have reinforced this notion. Although these findings suggest that a close relationship may exist between zinc homeostasis and the hematopoietic system, the evidence to date is derived from mature blood cells; thus, we addressed two key scientific questions, namely whether zinc plays a role in hematopoiesis, and—if so—which zinc transporter regulates this process. Using both zebrafish and mouse models, we found that HSCs require SLC39A10 for survival, and we provide compelling evidence that SLC39A10 is the master regulator of zinc upregulation in HSCs during early hematopoiesis.

## 2 Results

### 2.1 The Zinc Transporter *Slc39a10* is Required for Hematopoiesis in Zebrafish Embryos

**Figure 1:**
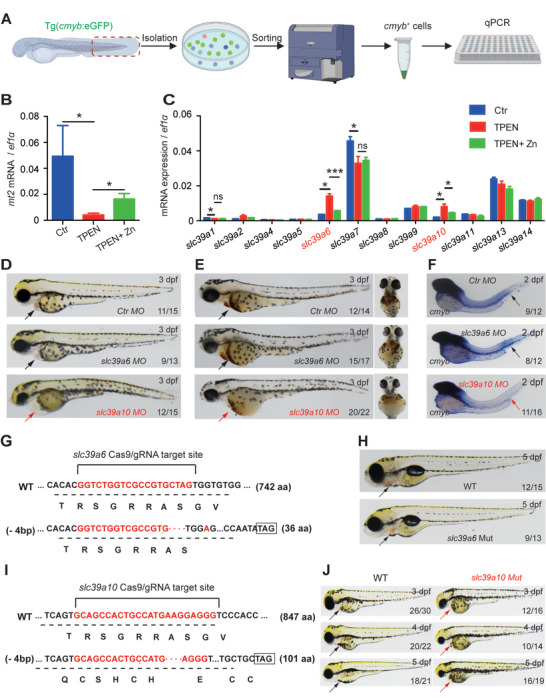
Functional screening in zebrafish reveals Sslc39a10 as a potentially important zinc transporter in early hematopoiesis. A) Schematic diagram depicting the strategy for performing functional screening in zebrafish. B,C) Relative levels of the indicated *mt2* and *slc39a* family genes were measured in *cmyb^+^* cells treated at 60–72 hpf with 100 µm TPEN or/and 100 µm ZnSO_4_ (*n* = 3 per group). D) Representative images of embryos at 3dpf following injection of a control morpholino (MO), *slc39a6* MO, or *slc39a10* MO. The arrows indicate the heart region. Note the visibly smaller head and presence of cardiac edema in the *slc39a10* morphant embryo. E) Representative images of o‐dianisidine‐stained embryos at 3dpf following injection of the control MO, *slc39a6* MO, or *slc39a10* MO. The arrows indicate the heart region. F) Whole‐mount in situ hybridization (WISH) of *cmyb* in 2dpf embryos after injection of the control MO, *slc39a6* MO, or *slc39a10* MO. Note the extremely low expression of *cmyb* in the caudal hematopoietic tissue (CHT) region (arrows) in the *slc39a10* morphant. G) DNA and corresponding amino acid sequences of the wild‐type (WT) *slc39a6* allele and the mutant allele after a 4‐nucleotide deletion (‐4 bp) using CRISPR/Cas9‐based editing, introducing a premature stop codon. H) Representative images of a WT embryo and *slc39a6* mutant sibling at 5dpf. The arrows indicate the heart region. I) DNA and corresponding amino acid sequences of the WT *slc39a10* allele and mutant allele after a 4‐nucleotide deletion (‐4 bp) using CRISPR/Cas9‐based editing, introducing a premature stop codon. J) Representative images of WT embryos and *slc39a10* mutant siblings at the indicated stages. The arrows indicate the heart region. The data in (B) and (C) are presented as the mean ± SD. *p* values were determined using one‐way ANOVA with Tukey's post hoc test (for multi‐group comparisons). *p < 0.05, ***p < 0.001, and ns, not significant.

To investigate the functional role of *slc39a* family members in embryonic hematopoiesis, we performed a functional screen in zebrafish embryos treated with the zinc chelator *N*,*N*,*N*′,*N*′‐Tetrakis (2‐pyridylmethyl) ethylenediamine (TPEN). Transgenic Tg(*cmyb*:eGFP) zebrafish, in which HSPCs express eGFP, were exposed to TPEN at 60–72 h post‐fertilization (hpf) in the absence or presence of zinc; *cmyb^+^* cells were then sorted, and mRNA levels were measured using qPCR (**Figure** **1**A). As shown in Figure 1B, the relative mRNA levels of *mt2*, a surrogate indicator of intracellular zinc,^[^ ^22^ ^]^ were significantly downregulated in the TPEN‐treated group, and this effect was significantly reduced by zinc supplementation, indicating that TPEN treatment decreases intracellular zinc levels in HSPCs in zebrafish embryos. Next, we measured the relative expression levels of various *slc39a* genes in *cmyb^+^* cells. We found that both *slc39a6* and *slc39a10* were upregulated following TPEN treatment, and this upregulation was significantly reduced by zinc supplementation (Figure 1C), suggesting that both *slc39a6* and *slc39a10* may participate in regulating zinc homeostasis in HSPCs in zebrafish embryos. Interestingly, we also found that *slc39a1* and *slc39a7* were downregulated following TPEN treatment, although zinc supplementation had no effect (Figure 1C). We, therefore, studied the role of *slc39a6* and *slc39a10* in hematopoiesis in zebrafish embryos.

Next, we microinjected morpholinos into zebrafish embryos in order to knock down the expression of either *slc39a6* or *slc39a10*. Notably, *slc39a10* morphants developed severe anemia based on o‐dianisidine staining (Figure 1D,E), accompanied by a clear reduction in HSPCs measured using whole‐mount in situ hybridization (WISH) for *cmyb* (Figure 1F); in contrast, these developmental changes were not observed in *slc39a6* morphants (Figure 1D,F). Similar results were obtained in zebrafish in which either *slc39a6* or *slc39a10* was knocked out using CRISPR/Cas9 (Figure 1G–J). We, therefore, focused on functionally characterizing the role of *slc39a10* in hematopoiesis in embryonic zebrafish.

### 2.2 *Slc39a10* Mutant Zebrafish have Increased Mortality and Impaired Hematopoiesis

**Figure 2:**
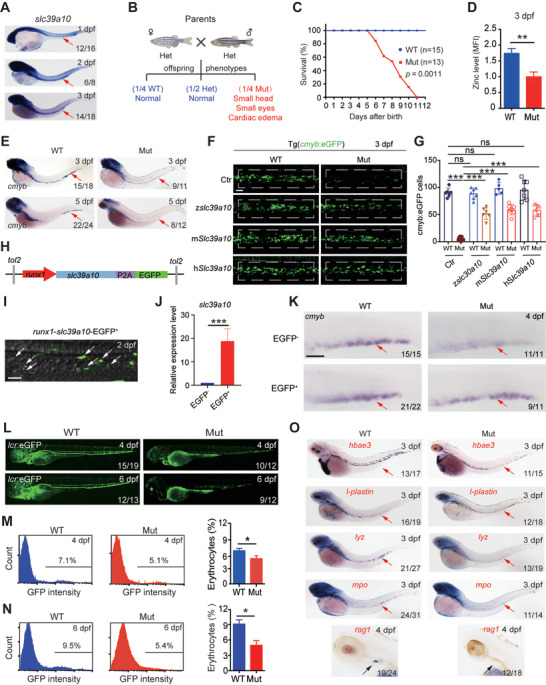
*Slc39a10* mutant zebrafish have defective hematopoiesis. A) WISH of *slc39a10* in WT embryos at the indicated stages. The arrows indicate a concentrated expression of *slc39a10* in the hematopoietic region. B) Mating strategy for producing offspring from *slc39a10* heterozygous crosses. The offspring were born at the expected Mendelian ratio. Note that the homozygous *slc39a10* mutant embryos have a small head, small eyes, and cardiac edema. C) Kaplan–Meier survival curve of WT and *slc39a10* mutant sibling (*n* = 15 and 13 embryos, respectively). D) Summary of zinc concentration measured in 3dpf WT and *slc39a10* mutant sibling embryos loaded with the fluorescent zinc probe FluoZin‐3, AM (*n* = 3 per group). E) WISH of *cmyb* in WT and *slc39a10* mutant siblings at the indicated stages. The arrows indicate the CHT region. F,G) Representative images of WT and Tg(*cmyb*:eGFP) *slc39a10* mutant sibling embryos either uninjected or injected with *slc39a10* mRNA of zebrafish, mouse, and human gene orthologs (F), and quantification of *cmyb^+^* cells in the CHT region at 3dpf (G). The dashed boxes indicated the region of HSPC counting. Scale bar, 50 µm. H) Schematic illustration of the construct of full‐length *slc39a10* driven by the *runx1* enhancer. I) Confocal imaging showing the expression of *Slc39a10* protein indicated by the green fluorescence (GFP) in *runx1* ^+^ HSPCs in the CHT region at 2 dpf. The white arrows indicated *runx1* ^+^ HSPC in the CHT. Scale bar, 50 µm. J) qPCR showing the mRNA level of *slc39a10* in EGFP^−^ and EGFP^+^ embryos at 4 dpf (*n* = 3 per group). K) WISH showing that the decreased expression of *cmyb* in *slc39a10* mutant embryos was partially rescued by overexpression of *slc39a10* in *runx1* ^+^HSPCs. The red arrows indicate *cmyb* ^+^ HSPCs in the CHT. Scale bar, 50 µm. L) Representative images of WT and *slc39a10* mutant sibling embryos in Tg(*globinLCR*:GFP) background at the indicated stages. M,N) Example FACS analysis plots of GFP fluorescence measured in erythrocytes obtained from WT and *slc39a10* mutant sibling embryos at the indicated stages and summary of the percentage of GFP^+^ erythrocytes (*n* = 4 per group). O) WISH of *hbae3*, *l‐plastin*, *lyz*, *mpo*, and *rag1* mRNA in WT and *slc39a10* mutant sibling embryos at the indicated stages. The red arrows indicate HSPCs in the CHT, and the black arrowheads indicate T cells in the thymus. Data in (D), (G), (J), (M), and (N) as mean ± SD. *p* values of survival in (C) were determined using the Log‐rank test, in (D), (G), (J), (M), and (N) using 2‐tailed unpaired Student's *t*‐test. *p < 0.05, **p < 0.01, ***p < 0.001, and ns, not significant.

To study the role of Sslc39a10 in hematopoiesis, we measured *slc39a10* expression during early development using WISH. We found that *slc39a10* was maternally expressed and could be detected in the blood island^[^ ^23^ ^]^ at 1dpf and in the CHT at 3dpf in wild‐type (WT) embryos (**Figure** **2**A and Figure S1, Supporting Information). We also found that s*lc39a10* mutant embryos were morphologically indistinguishable from WT siblings before 3dpf; however, starting at 3dpf, all mutants had a smaller head and eyes compared to WT siblings and died by 11dpf with cardiac edema (Figure 2B,C); similar results were obtained in *slc39a10* morphants. To examine whether the loss of *slc39a10* affected embryonic development by altering intracellular zinc levels, we used the zinc indicator FluoZin‐3 and found significantly lower zinc levels in *slc39a10* mutant zebrafish compared to WT siblings (Figure 2D). Together, these results support the notion that *slc39a10* is essential for embryonic development.

Next, we measured *cmyb* expression in 3dpf and 5dpf *slc39a10* mutant embryos and found a similar phenotype as in the *slc39a10* morphant embryos including a lack of HSPCs in the CHT (Figure 2E). To confirm that the *slc39a10* mutation underlies the loss of HSPCs, we performed a rescue experiment in which we microinjected WT *slc39a10* mRNA into *slc39a10* mutant embryos on the Tg(*cmyb*:eGFP) background. We found that overexpressing zebrafish *slc39a10* in mutant embryos partially—albeit significantly—increased the number of *cmyb^+^* HSPCs (Figure 2F,G), Besides, injection of human and mice *Slc39a10* mRNA into *slc39a10* mutants can also partly augment *cmyb^+^* HSPCs (Figure 2F,G), indicating that the loss of *slc39a10* underlies the hematopoietic defects and SLC39A10 is phylogenetically conserved among species. To further illustrate the HSPC‐specific role of *slc39a10* during HSPC development, we generated constructs with the *tol2* transposon to overexpress *slc39a10* or *mismatch*‐*slc39a10* fused with the EGFP reporter in HSPCs, which is driven by the *runx1* enhancer (Figure 2H–J and Figure S3A–C, Supporting Information). WISH showed that the increased expression of *slc39a10* in *runx1* ^+^ HSPC efficiently rescued the defects in HSPCs in *slc39a10* mutants and morphants (Figure 2K and Figure S3D, Supporting Information). By comparison, we performed endothelium cell (EC)‐specific overexpression of *slc39a10* or *mismatch*‐*slc39a10* fused with EGFP reporter driven the *fli1a* promoter (Figures S2A–C and S3E–G, Supporting Information). It was found that overexpression of *slc39a10* in ECs failed to reverse the decreased number of HSPCs in the CHT region in *slc39a10* mutants and morphants, respectively (Figures S2D and S3H, Supporting Information). These findings corroborate the HSPC‐autonomous but not the EC niche‐dependent roles of *Slc39a10* in controlling HSPC expansion.

Given that definitive HSPCs have the potential to give rise to all mature hematopoietic lineages, we then examined the hematopoiesis phenotype in detail by crossing the zebrafish to the Tg(*globinLCR*:GFP) background, in which erythrocytes express GFP.^[^ ^24^ ^]^ We found that the number of GFP‐positive cells was reduced in the *slc39a10* mutant embryos (Figure 2L), and FACS analysis revealed a slight but significant decrease in GFP‐positive cells in *slc39a10* mutant embryos compared to WT siblings at 4dpf (Figure 2M), with an even larger decrease at 6dpf (Figure 2N). Moreover, we found that the marker of embryonic erythrocytes, hemoglobin alpha embryonic‐3 *(hbae3*), was also reduced in the heart and CHT of *slc39a10* mutant embryos at 3dpf and 5dpf (Figure 2O and FigureS4, Supporting Information), suggesting that the loss of *slc39a10* leads to anemia in zebrafish. Notably, we also found that a variety of cell lineage markers, including *l‐plastin* (a marker of pan‐myeloid cells), *lyz* (a marker of monocytes), and *mpo* (a marker of neutrophils) were virtually undetectable in *slc39a10* mutant embryos at both 3dpf and 5dpf (Figure 2O and Figure S4, Supporting Information).^[^ ^25^ ^]^ Moreover, the expression of *rag1* (a marker of T cells) was also markedly decreased in *slc39a10* mutant embryos at 4dpf (Figure 2O), indicating that lymphoid development is also impaired. Taken together, these results suggest a complete lack of definitive hematopoiesis in *slc39a10* mutant embryos.

**Figure 3:**
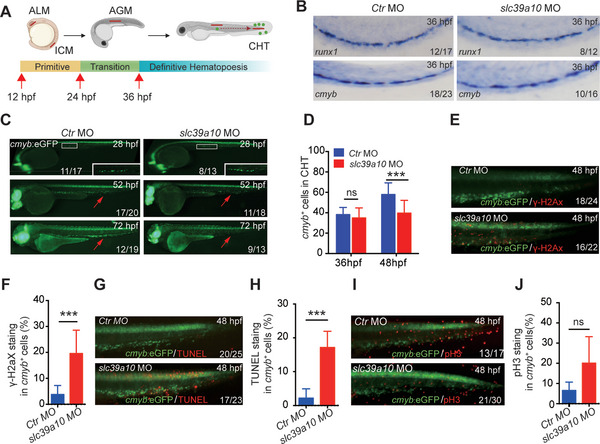
Loss of *slc39a10* reduces the number of HSPCs in zebrafish via increased cell death. A) Developmental timeline showing the sites of hematopoiesis in zebrafish. The primitive wave begins at 12 hpf in two locations, the anterior lateral mesoderm (ALM) and the intermediate cellular mass (ICM), generating the majority of monocytes and erythrocytes before 24 hpf. In the definitive wave, which begins at 36 hpf, HSPCs arise from the AGM region and then migrate to the CHT region, where they undergo transitional proliferation and differentiation. The red lines indicate the shifting sites of hematopoiesis, and the green dots indicate HSPCs. B) Representative brightfield images of the AGM region, showing WISH of *runx1* and *cmyb* in 36 hpf embryos injected with the control MO or the *slc39a10* MO. C,D) Example fluorescence images of 28, 52, and 72 hpf Tg(*cmyb*:eGFP) embryos injected with the control MO or *slc39a10* MO (C), and quantification of *cmyb^+^* cells in the CHT measured at 36dpf and 48 hpf (D; *n* = 26 for control and *n* = 29 for *slc39a10* morphants at 36 hpf, *n* = 35 for control and *n* = 40 for *slc39a10* morphants at 48 hpf). E,F) Representative images (E) and quantification (F) of *γ*‐H2aX immunostaining in the CHT region of 48 hpf Tg(*cmyb*:eGFP) embryos injected with the control MO or *slc39a10* MO (*n* = 10 each). G,H) Representative images (G) and quantification (H) of TUNEL immunostaining in the CHT region of 48 hpf Tg(*cmyb*:eGFP) embryos injected with the control MO or *slc39a10* MO (*n* = 18 for control and *n* = 21 for *slc39a10* morphants). I,J) Representative images (I) and quantification (J) of pH3 immunostaining in the CHT region of 48 hpf Tg(*cmyb*:eGFP) embryos injected with the control MO or *slc39a10* MO (*n* = 13 for control and *n* = 21 for *slc39a10* morphants). Data in (D), (F), (H), and (J) are presented as mean ± SD. *p* values in (D), (F), (H), and (J) using 2‐tailed unpaired Student's *t*‐test. ***p < 0.001 and ns, not significant.

Because the *slc39a10* morphant embryos and the *slc39a10* mutant embryos have a similar phenotype, and the mutant embryos are morphologically indistinguishable from WT siblings before reaching 3dpf, we measured the expression of several markers of primitive hematopoiesis in *slc39a10* morphant embryos and control siblings using WISH. In zebrafish, primitive hematopoiesis starts at 12 hpf in the anterior lateral mesoderm (ALM) and intermediate cellular mass (ICM), and then proceeds as a wave (**Figure** **3**A). We found that expression of the erythroid marker *gata1* ^[^ ^26^ ^]^ and the stem cell marker *scl* ^[^ ^27^ ^]^ was similar between *slc39a10* morphants and controls at the 16‐18‐somite stage (Figure S5, Supporting Information), suggesting that the loss of *slc39a10* does not affect primitive hematopoiesis, but appears to selectively affect definitive hematopoiesis.

### 2.3 Loss of *s* *lc39a10* Leads to DNA Damage and Cell Death in Zebrafish

To determine the mechanism underlying the reduced hematopoiesis observed in embryos lacking *slc39a10*, we examined the emergence, migration, proliferation, and cell death of HSPCs in *slc39a10*‐deficient embryos. We first analyzed the production of HSPCs in the AGM region and found that expression of the HSPC markers *runx1* and *cmyb* were similar at 36 hpf between *slc39a10* morphants and control embryos (Figure 3B), indicating that the emergence of HSPCs does not require *slc39a10*, which is consistent with that EC‐specific overexpression of *slc39a10* failed to rescue the hematopoietic phenotype. Next, given that HSPCs migrate to the CHT after their emergence in the AGM region, we used fluorescence imaging to track the migration of these HSPCs from 28 to 72 hpf in Tg(*cmyb*:eGFP) embryos (Figure 3C). We found that the number of *cmyb^+^* cells in the CHT was similar between *slc39a10* morphants and control embryos at 36 hpf, but was significantly reduced in the CHT of *slc39a10* morphants compared to control embryos at 48 hpf (Figure 3D), suggesting that the reduction in *cmyb* expression in the CHT of *slc39a10* morphant embryos occurs in this 12‐h window between 36 and 48 hpf. Finally, we measured DNA damage, cell death, and cell proliferation in the CHT at 48 hpf using *γ*‐H2AX, TUNEL, and phosphohistone H3 (pH3) staining, respectively. Compared to control embryos, we found significantly more DNA damage and cell death in the *slc39a10* morphant embryos (Figure 3E–H), but no significant difference in pH3 staining (Figure 3I,J). Taken together, these findings suggest that loss of *slc39a10* in zebrafish embryos impairs definitive hematopoiesis by increasing DNA damage and cell death in HSPCs.

### 2.4 Hematopoietic‐Specific Loss of *Slc39a10* Causes Impaired Hematopoiesis in Mice

**Figure 4:**
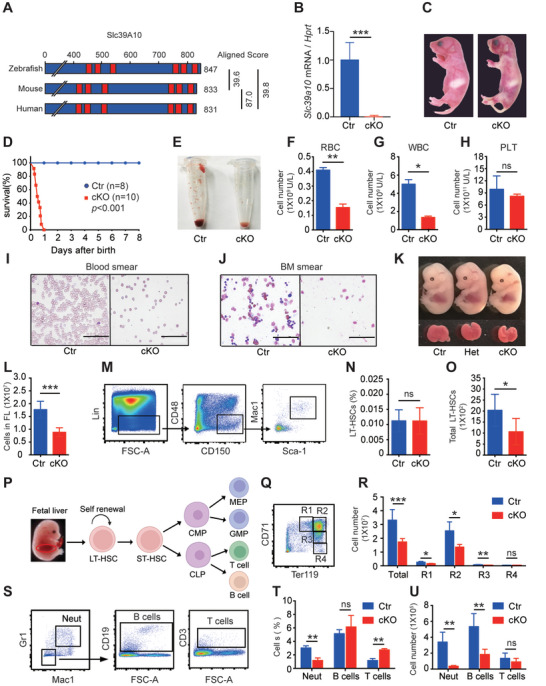
Mice lacking *Slc39a10* in hematopoietic cells have defective hematopoiesis and die within 1 day of birth. A) Alignment of the amino acid sequences of zebrafish (*Danio rerio*) *slc39a10*, mouse (*Mus musculus*) *Slc39a10*, and human (*Homo sapiens*) SLC39A10 proteins, as well as the scoring for protein homology obtained using ClustalW. B) *Slc39a10* mRNA levels were measured in FL LT‐HSCs obtained from control and *Slc39a10* cKO mice (*n* = 5 mice per group). C) Representative images of newborn (P1) control and cKO mouse pups. D) Kaplan–Meier survival curve of control and cKO mice (*n* = 8 and 10 for control and cKO, respectively). E) Example images of peripheral blood taken from control and cKO mice. F–H) Summary of red blood cell (RBC; F), white blood cell (WBC; G), and platelet (PLT; H) counts in the peripheral blood of control and cKO mice (*n* = 3 mice per group). I,J) Wright–Giemsa‐stained peripheral blood smears (I) and bone marrow smears (J) taken from control and cKO mice. Scale bars: 100 µm. K) Gross appearance of the whole body (top) and fetal liver (bottom) of control, heterozygous (het), and homozygous cKO embryos at E14.5. L) Absolute numbers of liver cells in control and cKO embryos measured at E14.5 (*n* = 10 and 7 for control and cKO, respectively). M) Fetal liver cells were obtained from E14.5 embryos, and long‐term repopulating HSCs (LT‐HSCs) were gated using flow cytometry. N,O) Summary of the percentage and absolute number of LT‐HSCs in the liver of control and cKO embryos (*n* = 10 and 7 for control and cKO, respectively). P) Schematic depiction of hematopoiesis showing the proliferation and differentiation of HSCs in the fetal mouse liver. Q,R) Gating strategy (Q) and absolute numbers (R) of the indicated erythrocyte developmental stages in the liver of control and cKO embryos were measured at E14.5 (*n* = 4 and 3 for control and cKO, respectively). S–U) Gating strategy (S), frequency (T), and absolute numbers (U) of myeloid cells (defined as Mac1^+^Gr1^+^), B cells (defined as CD19^+^), and T cells (defined as CD3^+^) in the liver of control and cKO embryos measured at E14.5 (*n* = 4 and 3 for control and cKO, respectively). Data in this figure are represented as mean ± SD. *p* values of survival in (D) were determined using the Log‐rank test, in (B), (F), (G), (H), (L), (N), (O), (R), (T), and (U) using 2‐tailed unpaired Student's *t*‐test. *p < 0.05, **p < 0.01, ***p < 0.001, and ns, not significant.

Given that SLC39A10 is phylogenetically conserved among zebrafish, mice, and humans (**Figure** **4**A and Figure S6, Supporting Information), and microarray data obtained from previous study^[^ ^28^ ^]^ suggest that *Slc39a10* is widely expressed throughout the mouse hematopoietic system (Figure S7, Supporting Information), we sought to examine the functional role of *Slc39a10* in definitive hematopoiesis in mice. Global *Slc39a10* knockout mice are embryonic lethal around E9.5 (Table S1, Supporting Information). In order to examine the phenotype in further detail, we then generated *Slc39a10* conditional knockout (cKO) mice with a *Vav‐Cre* line, which produces the Cre recombinase specifically in hematopoietic cells (*Slc39a10^fl/fl^;Vav‐Cre^+^*, referred to hereafter as cKO mice). Loss of *Slc39a10* expression in fetal liver HSCs (FL‐HSCs) was confirmed by a 95% reduction in *Slc39a10* mRNA levels in FL‐HSCs in cKO mice at E14.5 compared to control (*Slc39a10^fl/fl^*) littermates (Figure 4B). Although cKO offspring were born at the expected Mendelian ratio (data not shown), these mice developed hemorrhaging and pale skin (Figure 4C) and were early postnatal lethal, dying within 1 day of birth (Figure 4D). We also found that the peripheral blood collected from 1‐day‐old cKO mice was paler than in control mice (Figure 4E), and hematological analysis revealed that cKO pups had significantly reduced numbers of both red blood cells (Figure 4F) and white blood cells (Figure 4G), with no significant difference in platelets (Figure 4H). Moreover, both peripheral blood smears (Figure 4I) and bone marrow smears (Figure 4J) showed that the numbers of red blood cells, white blood cells, and other hematopoietic cell types were drastically reduced in cKO mice. In addition, a severe lack of red blood cells was observed in the heart, lungs, and liver of cKO pups (Figure S8, Supporting Information). Based on these findings, we conclude that cKO mice have severe anemia and developmental defects in hematopoiesis, affecting multiple lineages.

Interestingly, we also found that the cKO embryos were smaller in size at E14.5 compared to control littermates (Figure 4K), and we found a ≈50% reduction in total cell numbers in the FL of cKO embryos (Figure 4L). We then quantified the number of long‐term repopulating HSCs (LT‐HSCs, identified as Lin^−^Sca1^+^CD48^−^CD150^+^Mac1^+^ cells)^[^ ^9^, ^29^ ^]^ in the FL tissue of cKO and control embryos (Figure 4M). Although the percentage of LT‐HSCs at E14.5 was similar between cKO and controls (Figure 4N), the absolute number of LT‐HSCs was significantly lower in the cKO embryos (Figure 4O). HSCs are characterized by their ability to both self‐renew and differentiate into multiple lineages via short‐term HSCs to form a variety of mature blood cells including erythrocytes, monocytes, granulocytes, and lymphocytes (Figure 4P).^[^ ^30^ ^]^ We, therefore, characterized erythropoiesis in cKO embryos by performing flow cytometry of erythroid precursors using antibodies against the cell surface markers CD71 and Ter119 (Figure 4Q). We found that the total number of cells, as well as the numbers of pro‐erythroblasts (R1), basophilic erythroblasts (R2), and polychromatophilic erythrocytes (R3), were all significantly lower in cKO embryos compared to controls (Figure 4R). Moreover, cKO embryos had fewer granulocytes (defined as Mac1^+^Gr1^+^ cells) and fewer B cells (defined as CD19^+^ cells) in the FL compared to controls (Figure 4S–U). Together, these results indicate that the loss of *Slc39a10* results in severely impaired hematopoiesis.

### 2.5 Hematopoietic Stem Cells Derived from *Slc39a10* cKO Mice have Significantly Increased Apoptosis and Arrest at the G_1_ Phase

**Figure 5:**
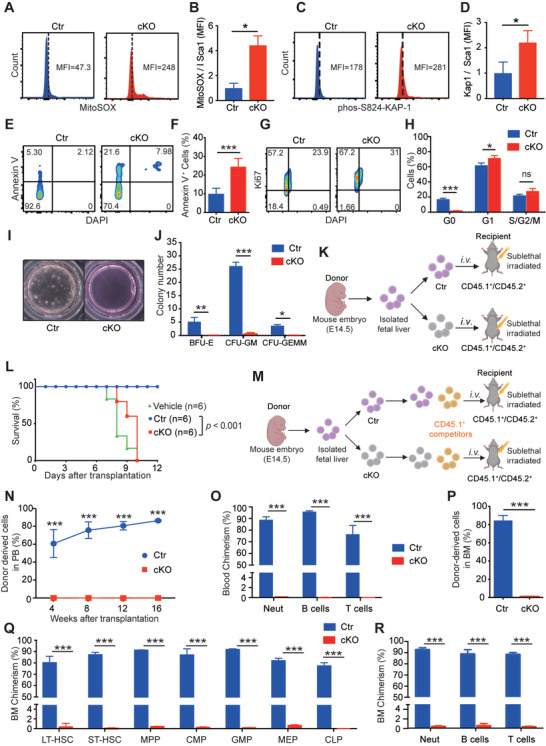
*Slc39a10*‐deficient HSCs have increased ROS levels, increased DNA damage, a higher prevalence of apoptosis, and impaired repopulation capacity. A,B) Representative FACS profiles (A) and quantification (B) of MitoSOX mean fluorescence intensity (MFI) measured in HSCs (Lin^−^Sca1^+^Mac1^+^) obtained from control and cKO embryos at E14.5 (*n* = 3 per group). C,D) Representative FACS profiles (C) and quantification (D) of intracellular p‐Kap1 staining in HSCs (Lin^−^Sca1^+^Mac1^+^) obtained from control and cKO embryos at E14.5 (*n* = 4 and 3 for control and cKO, respectively). E,F) Representative FACS profiles (E) and quantification (F) of annexin V staining in LT‐HSCs (Lin^−^Sca1^+^CD48^−^CD150^+^Mac1^+^) obtained from control and cKO embryos at E14.5 (*n* = 4 per group). G,H) Representative FACS profiles of intracellular Ki67 staining in HSCs (Lin^−^Sca1^+^Mac1^+^) obtained from control and cKO embryos at E14.5 (G), and summary of the percentage of cells in the indicated cell cycles (H; *n* = 3 per group). I,J) Representative images of in vitro colony assays performed using fetal liver cells obtained from control and cKO embryos at E14.5 (I), and summary of the number of primitive erythroid progenitor cells (BFU‐E), granulocyte‐macrophage progenitor cells (CFU‐GM), and multipotent granulocytes, erythroid, macrophage, and megakaryocyte progenitor cells (CFU‐GEMM) measured on day 12 (*n* = 3 per group). K) Schematic diagram depicting the transplantation strategy in which lethally irradiated CD45.1^+^/CD45.2^+^ recipient mice received donor‐derived (CD45.2^+^) fetal liver cells. L) Kaplan–Meier survival curve of CD45.1^+^/45.2^+^ mice that were lethally irradiated and then transplanted with vehicle or fetal liver cells obtained from either CD45.2^+^ control or cKO embryos (*n* = 6 recipients per group). M) Schematic diagram depicting the strategy for competitive transplantation in which lethally irradiated CD45.1^+^/CD45.2^+^ recipient mice received the indicated donor‐derived fetal liver (CD45.2^+^) cells and competitor (CD45.1^+^) cells at a 1:1 ratio. N) Time course showing the percentage of donor‐derived CD45.2^+^ cells in the peripheral blood of recipient mice measured at the indicated time points following transplantation (*n* = 5 recipients per group). O) Summary of the percentage of donor‐derived myeloid cells, B cells, and T cells in the peripheral blood of recipient mice 16 weeks after co‐transplantation with control or cKO cells (*n* = 5 recipients per group). P) Summary of the percentage of donor‐derived cells (CD45.2^+^) in the bone marrow of recipient mouse mice 16 weeks after transplantation (*n* = 5 recipients per group). Q) Summary of the percentage of donor‐derived LT‐HSCs, short‐term HSCs (ST‐HSCs), MPP (multipotent progenitor cells), CMP (common myeloid progenitor cells), GMP (granulocyte/monocyte progenitor cells), MEP (megakaryocyte/erythrocyte progenitor cells), and CLP (common lymphoid progenitor cells) 16 weeks after co‐transplantation with control or cKO donor cells (*n* = 5 recipients per group). R) Summary of the percentage of donor‐derived myeloid cells, B cells, and T cells in the bone marrow of recipient mice 16 weeks after co‐transplantation with control or cKO cells (*n* = 5 recipients per group). Data in this figure are represented as mean ± SD. *p* values of survival in (L) were determined using the Log‐rank test, in (B), (D), (F), (H), (J), (N), (O), (P), (Q), and (R) using 2‐tailed unpaired Student's *t*‐test. *p < 0.05, ***p < 0.001, and ns, not significant.

In zebrafish, we observed reduced numbers of HSPCs in *slc39a10* mutant embryos due to a significant increase in DNA damage and cell death. We, therefore, examined how the loss of *Slc39a10* in cKO mouse embryos affects the fate of HSCs. We first measured oxidative stress in HSCs using MitoSOX staining and found significantly increased levels of reactive oxygen species (ROS) in cKO HSCs compared to control littermates (**Figure** **5**A,B). In addition, the HSCs in cKO mice had significantly more DNA damage based on staining for p‐Kap1 (a marker of DNA damage)^[^ ^31^, ^32^ ^]^ (Figure 5C,D), and staining for annexin V (a marker of apoptosis) was significantly higher in cKO HSCs compared to controls (Figure 5E,F), suggesting that the lack of *Slc39a10* in HSCs leads to both DNA damage and apoptosis.

To determine whether the loss of *Slc39a10* affects the proliferation of HSCs, we performed cell cycle analysis of FL‐HSCs by staining for the nuclear marker Ki67 (to determine cell cycle) and DAPI (to measure DNA content) (Figure 5G). We found that compared to control embryos, a lower percentage of HSCs in cKO embryos were in the G_0_ phase, while a larger percentage of HSCs were arrested at the G_1_ phase, with no difference in other phases of the cell cycle (Figure 5H).

### 2.6 HSCs in cKO Mice have Impaired Repopulation Capacity

Given that cKO mouse embryos have reduced numbers of HSCs, we next performed a methylcellulose colony‐forming unit (CFU) assay to examine the repopulation capacity of FL‐HSCs in these mice. We found that burst‐forming unit erythroid (BFU‐E) cells, granulocyte‐macrophage cells (CFU‐GM), and multipotent progenitor cells (CFU‐GEMM) developed in control embryos but not in cKO embryos (Figure 5I,J), suggesting that *Slc39a10* is required for hematopoietic progenitor repopulation.

To further characterize the functional defects in HSCs lacking *Slc39a10*, we measured the hematopoietic reconstitution capacity of FL‐HSCs derived from cKO embryos and controls. To measure the cells' short‐term functional capacity, lethally irradiated congenic CD45.1^+^/CD45.2^+^ recipient mice received a transplant of either cKO FL cells or control FL cells (Figure 5K). We found that 100% of recipient mice that received either cKO FL cells or vehicle (i.e., no cells) died within 7–10 days, whereas 100% of mice that received control FL cells survived well beyond this time point (Figure 5L). Next, we performed a competitive transplantation experiment in order to determine the long‐term repopulation capacity of HSCs. We transplanted either cKO or control FL cells together with competitive WT CD45.1^+^ cells (at a 1:1 ratio) into lethally irradiated CD45.1^+^/CD45.2^+^ and analyzed the donor‐cell chimerism in the peripheral blood 4, 8, 12, and 16 weeks after transplantation (Figure 5M). We found that cKO FL‐HSCs failed to repopulate in the recipient animals' peripheral blood, whereas control FL‐HSCs contributed to ≈85% of the cells in the recipient animals' blood at 16 weeks (Figure 5N). Moreover, an analysis of peripheral blood and bone marrow cells from the recipient mice revealed severe defects in the multi‐lineage reconstitution capacity of donor‐derived cKO cells measured 16 weeks after transplantation (Figure 5O–R and Figure S9, Supporting Information). Together with the results obtained from the CFU assays, these experiments support our finding that the deletion of *Slc39a10* significantly impairs the reconstitution capacity of HSCs via a cell‐autonomous mechanism.

### 2.7 Zinc Deficiency Underlies the Impaired Hematopoiesis Observed in Both *Slc39a10*‐Deficient Zebrafish and *Slc39a10* cKO Mice

**Figure 6:**
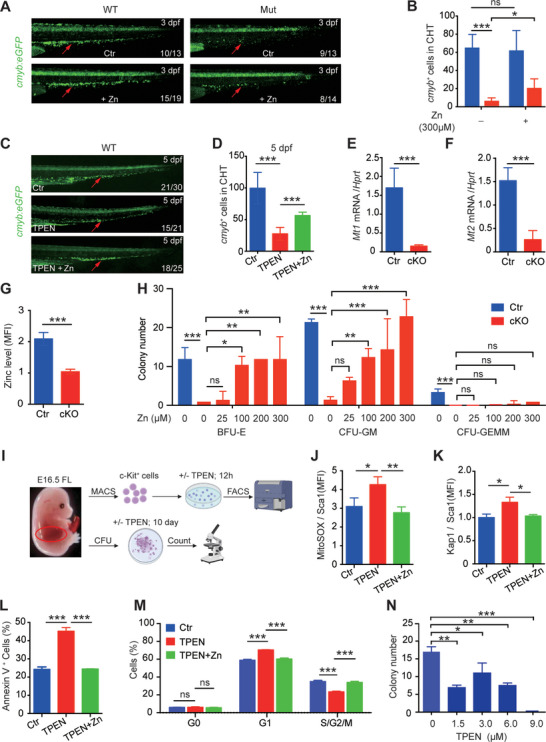
Zinc supplementation significantly reduces the impaired properties of HSPCs in *slc39a10* mutant zebrafish and HSCs in *Slc39a10* cKO mice. A,B) Representative images of the CHT region (arrows) in WT and *slc39a10* mutant sibling Tg(*cmyb*:eGFP) embryos in control conditions or exposed to 300 µm ZnSO_4_ (A), and quantification of *cmyb^+^* cells in the CHT region (B; *n* = 12 and 10 for wildtype sibling unexposed or exposed to 300 µm ZnSO_4_, *n* = 4 and 6 for *slc39a10* mutant unexposed or exposed to 300 µm ZnSO_4_). C,D) Representative images of the CHT region (arrows) in WT and *slc39a10* mutant sibling Tg(*cmyb*:eGFP) embryos treated with 100 µm TPEN and/or 100 µm TPEN+ZnSO_4_ (C), and quantification of *cmyb^+^* cells in the CHT region (D; *n* = 6, 12, and 8 for wildtype sibling unexposed or exposed to 100 µm TPEN and/or 100 µm TPEN+ZnSO_4_). E,F) Quantification of *Mt1* (E) and *Mt2* (F) mRNA measured in LT‐HSCs obtained from control and cKO mouse embryos at E14.5 (*n* = 5 per group). G) Summary of zinc concentration measured in LT‐HSCs obtained from control and cKO mouse embryos at E14.5 (*n* = 3 per group). H) In vitro colony assays were performed using fetal liver cells obtained from control and cKO embryos at E14.5 and from cKO fetal liver cells treated with ZnSO_4_ at the indicated concentrations (*n* = 3 per group). I) Schematic diagram depicting the strategy for in vitro cell culture of cKit^+^ cells obtained from WT fetal livers treated with or without TPEN. J–M) Summary of ROS levels (J), p‐Kap1 levels (K), the percentage of annexin V^+^ cells (L), and cell cycle distribution measured using Ki67 staining (M) in cKit^+^ cells obtained from WT mouse embryos at E16.5 and treated with 1.5 µm TPEN and/or zinc for 12 h (*n* = 3 per group). N) In vitro colony assays were performed using cKit^+^ cells obtained from WT embryos at E16.5 and treated with TPEN at the indicated concentration (*n* = 3 per group). Data in this figure are represented as mean ± SD. The data in (B), (E), (F), and (G) were analyzed using a 2‐tailed, unpaired Student's *t*‐test, and the data in (D), (H), (J), (K), (L), (M), and (N) were analyzed using a one‐way ANOVA with Tukey's post hoc test (for multi‐group comparisons). *p < 0.05; **p < 0.01; ***p < 0.001, and ns, not significant.

As a zinc transporter, the principal function of SLC39A10 is to import extracellular zinc into cells. Treating *slc39a10* mutant zebrafish with zinc increased the number of *cmyb^+^* cells in the CHT (**Figure** **6**A,B); in addition, treating Tg(*cmyb*:eGFP) zebrafish embryos with the chelator TPEN (100 µm) significantly reduced the number of *cmyb^+^* cells in the CHT, and this reduction was partially prevented by zinc supplementation (Figure 6C,D), suggesting that zinc deficiency directly causes impaired hematopoiesis in zebrafish. We then examined whether *Slc39a10* affects the survival of embryonic HSCs via its zinc importer activity in mice. Consistent with our data obtained for *slc39a10* mutant zebrafish, we found significantly lower levels of *Mt1* and *Mt2* mRNA in cKO FL‐HSCs compared to control FL‐HSCs (Figure 6E,F). In addition, FluoZin‐3 staining revealed significantly lower levels of zinc in cKO FL‐HSCs compared to control FL‐HSCs (Figure 6G).

Next, we examined whether the hematopoiesis defects in *Slc39a10* cKO mice could be rescued by zinc supplementation. Interestingly, we observed a dose‐dependent effect of zinc, with 300 µm zinc sufficient to nearly fully restore the defective colony‐forming capacity of cKO HSCs (Figure 6H). In contrast, adding 250 ppm zinc to the drinking water of pregnant females since conception^[^ ^33^ ^]^ failed to prolong the lifespan of their cKO pups, possibly due to the cKO embryos' inability to uptake zinc (Figure S10, Supporting Information). These findings are consistent with our zebrafish data and support the notion that SLC39A10 is the predominant zinc transporter in vertebrates with respect to controlling zinc influx in order to maintain the survival and functional properties of HSCs.

To test the pathological effects of zinc deficiency in WT embryonic HSCs, we isolated cells expressing cKit (a type III receptor tyrosine kinase expressed predominantly in bone marrow stem/progenitor cells) from the fetal liver of E16.5 C57BL/6 mice and cultured the cells in StemSpan medium (Figure 6I). After treatment for 12 h with the chelator TPEN, we measured ROS levels, DNA damage, apoptosis, and the cell cycle using flow cytometry. Consistent with our findings in *Slc39a10*‐deficient HSCs, we found that the TPEN‐treated cKit^+^ cells had higher ROS levels, increased DNA damage and apoptosis, and a higher prevalence of G_1_ phase arrest compared to control‐treated cells, and these effects were blocked by adding zinc to the TPEN‐containing medium (Figure 6J–M). In addition, our CFU assay experiments showed that TPEN‐treated cKit^+^ cells were significantly impaired at developing colonies (Figure 6N). Thus, under zinc‐deficient conditions, both WT zebrafish embryos and WT mice develop a hematopoietic phenotype similar to their corresponding hematopoietic‐specific *Slc39a10* knockout animals.

### 2.8 Loss of *p53*, *p21*, or *p16* Fails to Rescue Hematopoiesis in *Slc39a10* cKO Mice

**Figure 7:**
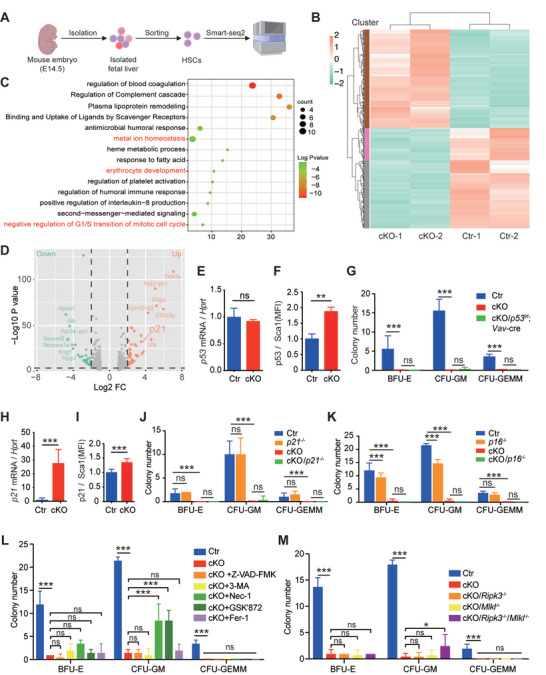
Inhibiting necroptosis partially restores the colony‐forming capacity of *Slc39a10‐*deficient HSCs. A) Schematic diagram depicting the strategy of sorting fetal LT‐HSCs from control and cKO embryos at E14.5 and performing RNA‐seq analysis. B) Heat map showing genes that are differentially expressed between fetal LT‐HSCs obtained from control and cKO mice; downregulation and upregulation (defined as a log2 fold change >1 or <−1 and *p* < 0.05) are shown in green and red, respectively. C) Gene Ontology (GO) analysis of the RNA‐seq data showing the top 15 enriched pathways in fetal LT‐HSCs between control and cKO mice (defined as a log2 fold change >1.3 or ←1.3 and *p* < 0.05). The pathways involved in metal ion homeostasis, erythrocyte development, and negative regulation of G1/S transition of the mitotic cell cycle are written in red. D) Volcano plot of the RNA‐seq data, showing the differentially expressed genes in fetal LT‐HSCs between control and cKO mice. The green and red dots indicate significantly downregulated and upregulated genes (defined as a log2 fold change >2 or <−2 and *p* < 0.05) respectively, while gray dots indicate genes that were not significantly upregulated or downregulated; the *p21* gene is indicated. E,F) Summary of normalized *p53* mRNA (E) and p53 protein (F) levels measured in LT‐HSCs obtained from control and cKO embryos at E14.5 (*n* = 5 and 3 for the mRNA and protein groups, respectively). G) In vitro colony assays were performed using fetal livers obtained from control, cKO, and *Slc39a10^fl/fl^*; *p53^fl/fl^*; *Vav‐Cre^+^* double‐knockout embryos at E14.5; colony numbers were measured on day 12 (*n* = 4 per group). H‐I, Summary of normalized *p21* mRNA (H) and p21 protein (I) levels measured in LT‐HSCs obtained from control and cKO embryos at E14.5 (*n* = 5 and 3 for the mRNA and protein groups, respectively). J,K) In vitro colony assays were performed using fetal livers obtained from control, cKO, cKO/*p21^−/‐^* (J), and cKO/*p16^−/‐^* (K) embryos at E14.5; colony numbers were measured on day 12 (*n* = 4 per group). L) Summary of CFUs formed by untreated control and cKO FL‐HSCs and cKO FL‐HSCs treated with 50 µm Z‐VAD‐FMK, 100 µm 3‐MA, 10 µm necrostatin‐1 (Nec‐1), 2.5 µm GSK’872, or 30 µm ferrostatin‐1 (Fer‐1) (*n* = 4 per group). M) Summary of CFUs formed by LT‐HSCs obtained from control, cKO, *cKO/Ripk3^−/−^*, *cKO/Mlkl^−/−^*, and *cKO*/*Ripk3^−/−^*/*Mlkl^−/−^* embryos at E14.5 (*n* = 3 per group). Data in this figure are represented as mean ± SD. The data in (E–I) were analyzed using a 2‐tailed, unpaired Student's *t*‐test, and the data in (J–M) were analyzed using a one‐way ANOVA with Tukey's post hoc test (for multi‐group comparisons). *p < 0.05; **p < 0.01; ***p < 0.001, and ns, not significant.

To explore the underlying molecular mechanisms in further detail, we performed RNA‐seq analysis on LT‐HSCs derived from cKO and control mice (**Figure** **7**A). The resulting heat map revealed several differentially expressed genes (defined as a log2 fold change >1 or <‐1 and *p* < 0.05) in fetal HSCs obtained from cKO mice compared to control HSCs (Figure 7B). Notably, we found that genes involved in the G_1_/S transition pathway were upregulated in cKO cells based on Gene ontology (GO) enrichment analysis (Figure 7C); in addition, *p21* (a p53‐targeted gene also known as *cdkn1a*) was significantly upregulated in cKO cells (Figure 7D). Moreover, the p53 signaling pathway was upregulated in cKO cells (Figure S11, Supporting Information). Interestingly, we found that although the levels of *p53* mRNA were similar between control and cKO cells (Figure 7E), p53 protein levels were significantly higher in cKO cells (Figure 7F), consistent with previous reports in *Slc39a10*‐deficient B cells^[^ ^34^ ^]^ and macrophages.^[^ ^17^ ^]^ Thus, we generated *Slc39a10^fl/fl^; p53^fl/fl^; Vav‐Cre^+^* double‐knockout mice and compared hematopoiesis with our *Slc39a10* cKO mice. We found that knocking out p53 in HSCs failed to rescue the cells' colony‐forming capacity (Figure 7G). Similarly, using our zebrafish model, we found that the hematopoietic defects in *slc39a10* mutant embryos were not rescued by injecting a *p53* MO, nor were the defects in *slc39a10* MO embryos rescued in *p53* mutant zebrafish (Figure S12, Supporting Information). Together, these results suggest that knocking out p53 is not sufficient to restore embryonic hematopoiesis in *Slc39a10‐*deficient animals.

We also found that the *p21* gene was significantly upregulated in cKO HSCs based on both qPCR (Figure 7H) and FACS analysis (Figure 7I). Given that the majority of HSCs derived from *Slc39a10* cKO mice were arrested in the G_1_ phase of the cell cycle, we hypothesized that *p21* and/or *p16*—both of which are critical G_1_ phase regulatory genes—might be involved in the hematopoietic defects observed in *Slc39a10‐*deficient HSCs. However, we found that knocking out either *p21* or *p16* in *Slc39a10* cKO mice failed to restore the ability of HSCs to develop colonies (Figure 7J,K).

### 2.9 Inhibiting Necroptosis in *Slc39a10* cKO HSCs Partially Rescues Hematopoiesis

Given that the loss of *Slc39a10* in cKO HSCs triggers cell death, we next performed rescue experiments using specific inhibitors of apoptosis (Z‐VAD‐FMK), autophagy (3‐AM), ferroptosis (ferrostatin‐1), and necroptosis (necrostatin‐1 and GSK’872). Interestingly, both necrosis inhibitors partially rescued the colony‐forming capacity of *Slc39a10‐*deficient HSCs, whereas all other inhibitors tested had no effect (Figure 7L and Figure S13, Supporting Information).

To further test this finding, we generated a *Ripk3*/*Mlkl* double‐knockout mouse, a genetic model with reduced susceptibility to necroptosis.^[^ ^35^, ^36^, ^37^ ^]^ We found that knocking out either *Ripk3* or *Mlkl* alone in *Slc39a10* cKO HSCs failed to restore the cells' colony‐forming capacity; importantly, however, knocking out both *Ripk3* and *Mlkl* partially rescued the colony‐forming capacity of *Slc39a10‐*deficient HSCs (Figure 7M), consistent with our results obtained with necrosis‐specific inhibitors.

## 3 Discussion

Here, we provide evidence that the zinc importer SLC39A10 plays a key role in hematopoiesis. Specifically, we show that SLC39A10‐mediated zinc import promotes the survival of HSCs during definitive hematopoiesis, thereby establishing a causal relationship between zinc homeostasis and embryonic hematopoiesis. With respect to the underlying mechanism, we found that zinc deficiency‐induced necroptosis contributes—at least in part—to the impaired properties of HSCs, implicating the SLC39A10‐zinc axis in the survival of fetal HSCs.

As a metal transporter, SLC39A10 has been shown to mediate the transport of zinc ions across the cell membrane into the cytoplasm in a variety of cell types including mouse macrophages,^[^ ^17^ ^]^ mouse B cells,^[^ ^34^ ^]^ and cells in the rat renal brush‐border membrane.^[^ ^16^ ^]^ Here, we report that SLC39A10 is the most responsive member of the zinc importer family to zinc deficiency in HSCs and is indispensable for the survival of these cells. Notably, our finding that zinc supplementation was sufficient to nearly fully rescue the colony‐forming capacity of *Slc39a10*‐deficient HSCs provides strong supporting evidence that HSCs require SLC39A10 to obtain sufficient levels of intracellular zinc for their survival.

It is well known that trace elements are essential for all living organisms, with iron playing a primary role in erythropoiesis. Indeed, we previously reported that transferrin receptor 1 (Tfr1) is required for fetal hematopoiesis by facilitating the uptake of iron at the cell surface by internalizing diferric transferrin.^[^ ^38^ ^]^ In addition, hematopoietic conditional *Tfr1* knockout mice have multiple lineage defects and die within 1 week of age, while Fe(III)/8‐hydroxyquinoline partially recovers the capacity of *Tfr1*‐deficient HSCs. Here, we report that zinc appears to play an even more important role than iron in fetal HSCs, with iron likely playing a preferential role in megakaryocyte‐erythroid progenitors.

Our finding that a loss of *Slc39a10* in HSCs leads to significantly increased levels of ROS, DNA damage, apoptosis, and cell cycle arrest in the G_1_ phase is consistent with previous reports that low intracellular zinc levels induce oxidative DNA damage and DNA repair‐related gene expression in several cell lines.^[^ ^39^, ^40^, ^41^ ^]^ In addition, several studies suggest that depleting intracellular zinc can cause apoptosis and cell cycle arrest in a variety of cell types.^[^ ^42^, ^43^, ^44^ ^]^ Similarly, we found that treating wild‐type fetal HSCs with the chelator TPEN reduced the number of HSCs via necroptosis. Thus, our findings provide compelling evidence to support the notion that the SLC39A10‐zinc axis plays key roles both in the survival of HSCs and in early hematopoiesis. Moreover, our results indicate that SLC39A10 is a survival factor for fetal HSCs and may function as a sensor to maintain zinc homeostasis during definitive hematopoiesis.

We previously reported that knocking out *p53* expression largely rescued the phenotype in macrophage‐specific *Slc39a10*‐knockout mice upon LPS stimulation.^[^ ^17^ ^]^ Interestingly, in the present study, we found that although the p53 signaling pathway was significantly upregulated in our *Slc39a10* cKO mice, knocking out p53 failed to restore hematopoiesis in either *Slc39a10* cKO mice or *slc39a10* mutant zebrafish, indicating that the loss of Slc39a10 may induce a p53‐independent form of cell death in HSCs. Moreover, knocking out either *p21* or *p16* also failed to restore the survival of *Slc39a10*‐deficient HSCs.

Interestingly, we found that inhibiting necroptosis in mice—either pharmacologically or using genetic knockout strategies—partially rescued the repopulation capacity of *Slc39a10*‐deficient HSCs, consistent with a previous report that activating the necroptosis pathway impairs hematopoiesis.^[^ ^45^ ^]^ Recently, Yamashita and Passegue showed that a TNF‐*α*‒mediated pro‐survival mechanism prevents necroptosis in HSCs.^[^ ^46^ ^]^ Moreover, acute loss of *Ripk1* and hematopoiesis‐specific *Ripk1* knockout mice led to bone marrow failure, which can be partially rescued by loss of receptor‐interacting serine/threonine‐protein kinase 3 (RIPK3) or mixed lineage kinase domain‐like pseudokinase (MLKL), indicating that RIPK1‐deficient hematopoietic cells partly undergo RIPK3‐mediated necroptosis.^[^ ^47^, ^48^ ^]^ Although MLKL is believed to be immediately downstream of RIPK3, several studies^[^ ^49^, ^50^ ^]^ revealed that Mlkl and Ripk3 have distinct functions in the necroptotic signaling pathway. It is therefore interesting to note that in our study, knocking out either *Ripk3* or *Mlkl* failed to restore the survival of *Slc39a10*‐deficient HSCs, and knocking out both *Ripk3* and *Mlkl* only partially restored the functional properties of *Slc39a10*‐deficient HSCs, reflecting the complexity of necroptotic signaling during hematopoiesis. Taken together, these results indicate that zinc deficiency‐medicated cell death may be unique to fetal HSCs, thus highlighting the important role that necroptosis plays in regulating hematopoiesis.

## 4 Conclusion 

In conclusion, we report that SLC39A10 has an essential function in fetal definitive hematopoiesis. Our findings provide compelling evidence supporting the notion that zinc homeostasis is critical for maintaining hematopoiesis and the development of early HSCs. By mediating zinc homeostasis, SLC39A10 is a survival factor for fetal HSCs and may serve as a novel target in developing new stem cell‐based therapeutic strategies. Further studies are clearly warranted in order to explore the potential therapeutic applications of these findings, particularly in zinc deficiency‐related anemia, immune deficiency, and other related diseases.

## 5 Experimental Section

### Zebrafish and Mouse Strains

Zebrafish strains, including the wild‐type Tubingen strain, Tg(*globinLCR*:GFP), Tg(*cmyb*:eGFP), *slc39a10*, *slc39a6*, and *p53* mutant strains, were raised in an incubator at 28.5 °C. Heterozygous *Slc39a10^fl/+^* knockout mice were bred with *Vav‐Cre* transgenic mice to generate *Slc39a10^fl/fl^;Vav‐Cre^+^* cKO mice in which *Slc39a10* was deleted selectively in HSCs and all downstream hematopoietic lineages; the morning in which a vaginal plug was observed in the female was defined as embryonic day 0.5 (E0.5). All animal experiments were approved by the Institutional Animal Care and Use Committee of Zhejiang University (Reference No.: 14 356).

### Functional Screening

Embryos on the Tg(*cmyb*:eGFP) background were exposed to 100 µm of the zinc chelator TPEN at 60–72 hpf in the absence or presence of 100 µm ZnSO_4_. The CHT regions were then cut into pieces and dissociated completely by trituration to form a single‐cell suspension. The suspended cells were then washed two times with Hank's buffered salt solution (HBSS; Invitrogen) and filtered through a 40‐µm cell strainer. The cells were then sorted using a FACS ARIA II SORP cell sorter (BD Biosciences). After sorting, the cells were directly homogenized in TRIzol LS reagent (Invitrogen), and total RNA was extracted using the Direct‐zol RNA kit (R2050, Zymo Research). The sequences of the primers for quantitative RT‐PCR are listed in Table S4, Supporting Information.

### Morpholinos, mRNA Synthesis, Vector Construction, and Microinjection

*slc39a10* mRNA was targeted by injecting a translational start codon morpholino (5′‐GTGTGAACTCTCATCATCTCCTCTC‐3′) into one‐cell stage embryos. The *slc39a6* MO sequence was 5′‐CAACATCATTCAC TGCTTACCGGGA‐3′, the *p53* MO sequence was 5′‐GCGCCATTGCTTTGCAAGAATTG‐3′, and the control MO sequence was 5′‐CCTCTTACCTCAGTTACAATTTATA‐3′. mRNAs of zebrafish *slc39a10*, mouse *Slc39a10*, and human *SLC39a10* cDNA were cloned and inserted into the pCS2 expression plasmid and were synthesized using mMESSAGE mMACHINE mRNA transcription synthesis kit (Invitrogen; AM1344), and cleaned up using RNAclean Kit (TIANGEN; DP412), respectively. 100 ng of mRNAs were injected into the one‐cell stage embryos. For the HSPC and EC‐specific overexpression experiments, the full‐length cDNA of *slc39a10* was cloned and assembled into the pDestTol2pA2 vector with a *runx1* enhancer or *fli1a* promotor, and an EGFP reporter to generate pDestTol2pA2‐*runx1*‐*slc39a10*‐v2a‐EGFP, pDestTol2pA2‐*runx1*‐*mis*‐*slc39a10*‐v2a‐EGFP, pDestTol2pA2‐*fli1a*‐*slc39a10*‐v2a‐EGFP, and pDestTol2pA2‐*fli1a*‐*mis*‐*slc39a10*‐v2a‐EGFP plasmid. The plasmid (50 pg) and Tol2 mRNA (25 pg) were co‐injected into one cell‐stage zebrafish embryo at the yolk/blastomere boundary.

### Whole‐Mount In Situ Hybridization

WISH was performed as described previously,^[^ ^51^ ^]^ with slight modifications, and images were captured using a Nikon SMZ18 stereo microscope.

### o‐Dianisidine Staining

Before staining, a stock solution of 100 mg o‐dianisidine dissolved in 70 mL ethanol was prepared at 4 °C in the dark. For staining, live embryos were exposed to an o‐dianisidine working solution consisting of 2 mL o‐dianisidine stock solution, 0.1 m sodium acetate (pH 4.5), and 0.65% H_2_O_2_ in a small glass bottle for 3–5 min at room temperature. To stop the staining process, the embryos were washed two times in phosphate‐buffered saline (PBS) and were imaged using a Nikon SMZ18 stereo microscope.

### Generation of Zebrafish Knockout Lines Using CRISPR/Cas9

Target sites were designed and selected using the CHOPCHOP website (https://chopchop.cbu.uib.no/). Guide RNA (gRNA) templates were synthesized as previously described,^[^ ^52^ ^]^ and gRNAs were mixed with Cas9 protein (M0646T, New England Biolabs) for microinjection into one‐cell stage embryos. T7 endonuclease 1 was used to evaluate the efficiency of target gene disruption, and F0 embryos with the highest editing efficiency were raised. Heterozygous F1 fish were identified using DNA sequencing of the offspring of F0 fish outcrosses. The sequences of the genotyping primers are listed in Table S2, Supporting Information.

### FACS Analysis to Measure the Number of Erythrocytes

Embryos at 4dpf and 6dpf on the Tg(*lcr*:eGFP) background were crushed in HBSS, followed by complete dissociation to a single‐cell suspension by trituration. Next, the cells were washed two times with HBSS and filtered with a 40‐µm cell strainer. Finally, the cells were harvested in HBSS and examined using a Cytomics FC 500 MCL flow cytometer (Beckman Coulter, Inc.).

### Immunofluorescence and TUNEL Staining

Embryos were fixed in 4% paraformaldehyde at 4 °C overnight. After fixation, the embryos were washed three times for 10 min in PBS and then incubated in a 1:20 dilution of trypsin (stock concentration: 0.25%) in PBS at room temperature for 15 min. Precooled acetone was then added to the samples, which were then incubated at −20 °C for 7 min. The samples were then washed three times 5 min in PBS and then blocked in PBS containing 10% fetal bovine serum (FBS) for 0.5 h. The samples were then incubated in primary antibodies dissolved in PBS containing 2% serum at 4 °C overnight and then washed five times 20 min in PBS. Secondary antibodies dissolved in PBS containing 2% serum were added to the sample, followed by a wash with PBS. For *γ*‐H2AX immunostaining, anti‐phospho‐Histone H2AX (Ser139) (05‐636‐I, EMD Millipore) and Alexa Fluor 555‐conjugated goat anti‐rabbit (A‐21428, Invitrogen) were used as the primary and secondary antibodies, respectively. For phospho‐histone H3 (pH3) immunostaining, anti‐pH3‐Ser‐10 (sc‐8656‐R, Santa Cruz Biotechnology) and Alexa Fluor 555‐conjugated goat anti‐rabbit (A‐21428, Invitrogen) were used as the primary and secondary antibodies, respectively. The TUNEL assay was performed using the fluorescein‐based In Situ Cell Death Detection Kit (12156792910, Roche) in accordance with the manufacturer's instructions. Note that *cmyb* fluorescence was quenched after fixation, and anti‐GFP (A‐11120, Invitrogen) and Alexa Fluor 488‐conjugated goat anti‐mouse (A‐10680, Invitrogen) antibodies were used as the primary and secondary antibodies, respectively, for the aforementioned immunofluorescence and TUNEL staining. The images were analyzed using the Multi‐point tool in ImageJ.

### Measurement of Zinc Levels Using Fluozin‐3, AM

The cell membrane‐permeable fluorescent zinc indicator FluoZin‐3, AM (F24195, Invitrogen) was used as described previously to measure intracellular zinc concentration.^[^ ^17^, ^53^ ^]^ In brief, the embryos were anesthetized and washed three times for 5 min in PBS. The embryos were then crushed to form a single‐cell suspension and suspended in 200 µL detection buffer consisting of 5 mm glucose, 1 mm MgCl_2_, 1 mm NaH_2_PO_4_, 1.3 mm CaCl_2_, 25 mm HEPES, 120 mm NaCl, 5.4 mm KCl, and 1 µm FluoZin‐3, AM (pH 7.5) for 30 min at 37 °C. Following FluoZin‐3 loading, the cells were analyzed as described previously.^[^ ^54^

### PCR Genotyping of Transgenic Mice

Heterozygous *Slc39a10^fl/+^* knockout mice were bred with *Vav‐Cre* transgenic mice to generate *Slc39a10^fl/fl^;Vav‐Cre^+^* cKO mice in which *Slc39a10* was deleted selectively in HSCs and all downstream hematopoietic lineages. For genotyping transgenic mice, tail‐tip biopsies were used to extract genomic DNA for PCR analysis using the primers listed in Table S5, Supporting Information.

### Blood and Bone Marrow Smears

Blood and bone marrow smears were prepared from control and cKO pups by air drying and were stained with Wright‐Giemsa.

### Histological Analysis

Control and cKO pups were fixed in 4% paraformaldehyde and then embedded in paraffin. Using a microtome (RM2235, Leica), 5‐µm sections were prepared and stained with hematoxylin and eosin for histological examination.

### Hematological Parameters

Peripheral blood was obtained from control and cKO pups and analyzed using an ADVIA 2120i hematology analyzer (Siemens) at the Center for Drug Safety Evaluation and Research, Zhejiang University.

### Flow Cytometry

Fetal livers were isolated from mouse embryos at E14.5 under a dissecting microscope, and cell suspensions were prepared in PBS containing 2% FBS by repeatedly flushing through needles ranging from 18‐ to 27‐gauge. The cells were then passed through a nylon mesh with a pore size of 70 µm, and red blood cells were lysed using RBC lysis buffer (00‐4333‐57, eBioscience).

For staining LT‐HSCs, the cells were first incubated with Fc Block followed by biotin‐conjugated lineage marker antibodies (CD3e, CD4, CD5, CD8a, B220, Gr‐1, Ter119, and CD11b), followed by APC‐Cy7‐conjugated streptavidin, PE‐Cy7‐conjugated Sca‐1, APC‐conjugated CD11b, PE‐conjugated CD150, and Percp‐Cy5.5‐conjugated CD48 antibodies.^[^ ^9^ ^]^ All antibodies were purchased from eBioscience or BioLegend. FACS data were analyzed using FlowJo software.

### ROS, DNA Damage, Apoptosis, and Cell Cycle Status Analysis

To measure ROS, cells were first labeled with surface markers (Lin^−^Sca1^+^Mac1^+^) to identify the relevant HSC subpopulation, and then incubated at 37 °C for 30 min in PBS containing 10 µm MitoSOX Red (M36008, Invitrogen). The cells were then washed twice with PBS, followed by flow cytometric analysis.

p‐Kap1 was reported as a marker for DNA damage.^[^ ^31^, ^32^ ^]^ Therefore, cells were first labeled with surface markers (Lin^−^Sca1^+^Mac1^+^), fixed, and then permeabilized. The cells were then stained with a p‐Kap1 antibody and Alexa Fluor 488‐conjugated goat anti‐rabbit antibody (A‐10680, Invitrogen) as the primary and secondary antibodies, respectively. The cells were then washed twice with PBS, and fluorescence was measured using flow cytometry.

To measure apoptosis, the cells were first labeled with surface markers (Lin^−^Sca1^+^CD48^−^CD150^+^Mac1^+^), and then immunostained for annexin V (MultiSciences) and DAPI in accordance with the manufacturer's instructions.

For intracellular Ki67 staining, the cells were first labeled with surface markers (Lin^−^Sca1^+^Mac1^+^), fixed, and permeabilized. The cells were then stained with an Alexa Fluor 488‐conjugated Ki67 antibody (652 417, BioLegend) at 37 °C for 30 min. DAPI (422 801, BioLegend) was added to a final concentration of 20 µg mL^−1^ immediately before flow cytometry.

### Transplantation Experiments

For competitive transplantation, 1 × 10^5^ nucleated donor cells (CD45.2^+^) obtained from E14.5 WT and cKO fetal livers were mixed with 1 × 10^5^ freshly isolated bone marrow nucleated cells (BMNCs) (CD45.1^+^), and the mixture was injected intravenously via the caudal vein into CD45.1^+^/CD45.2^+^ mice that were lethally irradiated (dose: 9 Gy). Peripheral blood cells were obtained from the recipients 4, 8, and 12 weeks after transplantation and stained with CD45.1‐PE and CD45.2‐APC for flow cytometric analysis; 16 weeks after transplantation, bone marrow and peripheral blood were collected for flow cytometric analysis. For the lethal transplantation experiments, 2 × 10^5^ nucleated donor cells (CD45.2^+^) obtained from E14.5 WT and cKO fetal liver were injected intravenously via the caudal vein into lethally irradiated CD45.1^+^/CD45.2^+^ recipient mice.

### Measurements of Zinc Levels in LT‐HSCs Using FluoZin‐3, AM

Fetal livers were isolated from E14.5 embryos under a dissecting microscope, and cells were suspended in the detection buffer. The fetal liver cells were first incubated with Fc Block and then labeled with surface markers (Lin^−^Sca1^+^CD48^−^CD150^+^Mac1^+^) to identify the relevant LT‐HSC subpopulation. The cells were then suspended in 200 µL detection buffer containing 1 µm FluoZin‐3, AM (pH 7.5) for 30 min at 37 °C. After washing several times with detection buffer, each sample was then adjusted to 500 µL with detection buffer and analyzed using flow cytometry.

### Measurements of p53 and p21 Protein Levels in LT‐HSCs Using FACS

For staining intracellular p53 and p21, cells were first labeled with surface markers (Lin^−^Sca1^+^CD48^−^CD150^+^Mac1^+^), fixed, and permeabilized. The cells were then stained with a p53 (9282s, Cell Signaling) or p21 (2947t, Cell Signaling) primary antibody, followed by an Alexa Fluor 488‐conjugated goat anti‐rabbit (A‐10680, Invitrogen) secondary antibody. The cells were then washed twice with PBS and analyzed using flow cytometry.

### In Vitro Methylcellulose Colony‐Forming Assays

Fetal liver suspensions were prepared in Iscove's Modified Dulbecco's Medium (IMDM) supplemented with 2% FBS, and red blood cells were lysed using an RBC lysis buffer. A total of 3 × 10^4^ FL cells were seeded in MethoCult M3434 (03434, STEMCELL Technologies) containing recombinant mouse stem cell factor (SCF; 50 ng mL^−1^), recombinant mouse IL‐3 (10 ng mL^−1^), recombinant human IL‐6 (10 ng mL^−1^), and erythropoietin (U mL^−1^). All assays were performed in triplicate. Colonies were counted after 12 days of incubation at 37 °C in humidified air containing 5% CO_2_.

For methylcellulose colony‐forming assays using zinc supplementation or specific inhibitors of cell death pathways, the fetal liver suspensions were prepared as described above, and 1 × 10^4^ FL cells were seeded in MethoCult M3434 containing zinc or specific inhibitors of cell death pathways at the indicated concentrations.

### SMART‐Seq2 Analysis of Fetal LT‐HSCs

Fetal livers were isolated from control and cKO embryos at E14.5, and cell suspensions were prepared in PBS containing 2% FBS. The cells were then labeled with surface markers as described above (Lin^−^Sca1^+^CD48^−^CD150^+^Mac1^+^). After sorting using a FACS ARIA II SORP cell sorter (BD Biosciences), fetal LT‐HSCs were directly collected in PBS containing 20% FBS, collected by centrifugation at 3000 rpm for 30 min at 4 °C, resuspended in cell lysis buffer, and analyzed as described previously.^[^ ^55^ ^]^ Sample reads were aligned to the Ensembl *Mus musculus* genome GRCm38.91 (https://www.ensembl.org/) using the HISAT package. The mapped reads of each sample were assembled using StringTie, and all transcriptomes were merged to reconstruct a comprehensive transcriptome using Perl scripts. After the final transcriptome was generated, StringTie and edgeR were used to estimate the expression levels of each transcript. StringTie was then used to calculate FPKM (Fragments Per Kilobase of transcript per Million mapped reads) in order to quantify the mRNAs. Differentially expressed mRNAs and genes were selected based on a log2 fold change >1.3 or <−1.3 and *p* < 0.05 using *R*. GO enrichment analysis for the differentially expressed genes was performed using topGO with the parentChild algorithm. The top 15 enriched biological processes with an adjusted *p*‐value <0.01 (Fisher's exact test) were selected for further analysis using the RT‐PCR primers listed in Table S6, Supporting Information.

### Isolation and Culture of Mouse HSCs

Fetal livers were isolated from WT mouse embryos at E16.5, and cell suspensions were prepared in PBS containing 2% FBS followed by RBC lysis. The cells were then labeled with a biotin‐conjugated cKit antibody at 4 °C for 30 min. After washing with magnet buffer, the cells were then incubated in biotin‐bead buffer at 4 °C for 30 min. After adding 2 mL magnet buffer, the cells were transferred to a magnetic frame, and cKit^+^ cells were attached to the inside of the magnetic frame. The cKit^+^ cells were then cultured in StemSpan SFEM Medium (09650, STEMCELL Technologies) containing 50 ng mL^−1^ mouse SCF (PeproTech), 20 ng mL^−1^ mouse FMS‐like tyrosine kinase 3 ligand (PeproTech), and 10 ng mL^−1^ mouse thrombopoietin (PeproTech) at 37 °C in 5% CO_2_ as described previously.^[^ ^56^

### Statistical Analysis

Flow cytometry results were analyzed by flowJo version 10.8.1. The fluorescent images were rectangle, image>adjust>brightness/contrast, and analyzed using the Multi‐point tool in ImageJ. Data were analyzed and plots were generated using GraphPad Prism version 7.0, and all summary data are presented as the mean ± standard deviation (SD). The sample size for each statistical analysis is provided in the figure legends. Where indicated, differences between groups were analyzed using a 2‐tailed, unpaired Student's *t*‐test (for comparing two groups) or one‐way ANOVA with Tukey's post hoc test (for multi‐group comparisons), and the log‐rank test was used to analyze the survival curves. Differences with a *p*‐value of <0.05 were considered statistically significant.

## Conflict of Interest

The authors declare no conflict of interest.

## Author Contributions

X.H., C.G., and J.X. contributed equally to this work. J.M., F.W., X.H., and C.G. designed the experiments. X.H. and C.G. acquired and analyzed the data. J.X. performed the whole body and specific‐tissue rescue experiment in zebrafish. Z.X. performed the CRISP/Cas9‐mediated *slc39a10* and *slc39a6* gene knockout experiments. S.H. and R.W. assisted in some murine experiments. P.X. and T.C. helped experiment with zebrafish. X.H., C.G., J.X., J.M., and F.W. drafted and revised the manuscript. J.M., F.W., L.Z., and J.P. obtained funding and supervised the study. All authors approved the final version of the paper.

## Supporting information

Supporting Information

## Data Availability

The data that support the findings of this study are available from the corresponding author upon reasonable request.
