# Supplementary material for: The Zinc Transporter SLC39A10 Plays an Essential Role in Embryonic Hematopoiesis
Source: Adv Sci (Weinh). 2023 Apr 17;10(17):2205345. doi: 10.1002/advs.202205345 (PMC10265086; doi:10.1002/advs.202205345)
Supplement: Supplementary file 1 — Supporting Information [file ADVS-10-2205345-s001.pdf]

ADVANCED SCIENCE
Open Access

Supporting Information

for *Adv. Sci.*, DOI 10.1002/advs.202205345

The Zinc Transporter SLC39A10 Plays an Essential Role in Embryonic Hematopoiesis

Xuyan He, Chaodong Ge, Jun Xia, Zhidan Xia, Lu Zhao, Sicong Huang, Rong Wang, Jianwei Pan, Tao Cheng, Peng-Fei Xu, Fudi Wang and Junxia Min**

Supporting Information

The Zinc Transporter SLC39A10 Plays an Essential Role in Embryonic Hematopoiesis

Xuyan He, Chaodong Ge, Jun Xia, Zhidan Xia, Lu Zhao, Sicong Huang, Rong Wang, Jianwei Pan, Tao Cheng, Peng-Fei Xu, Fudi Wang and Junxia Min

This file includes:

Supplementary Figures S1-S13

Supplementary Tables S1-S8

Supplementary Figures

Figure S1

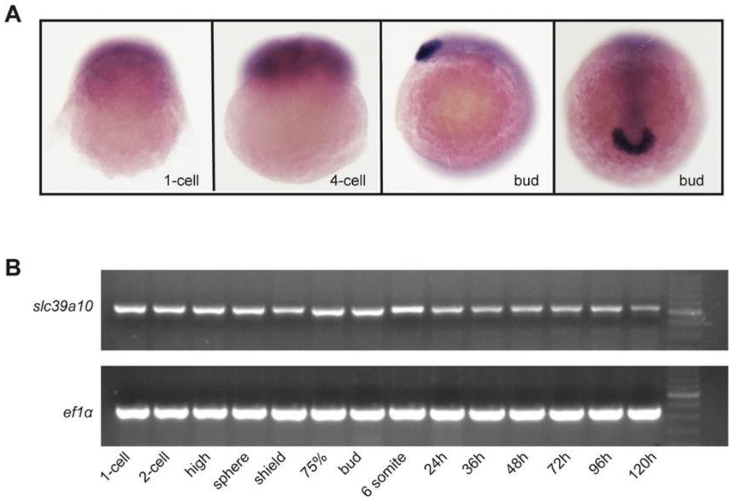

Figure S1. *slc39a10* is maternally expressed in zebrafish. A, *In situ* hybridization of wild-type zebrafish embryos using the *slc39a10* probe at the indicated embryonic stage. Note the expression of *slc39a10* at the one-cell stage and the concentrated expression in the polster at the bud stage. **B**, Semi-quantitative RT-PCR of *slc39a10* mRNA in wild-type embryos at the indicated stages; times (e.g., “24h”) refer to hours postfertilization.

Figure S2

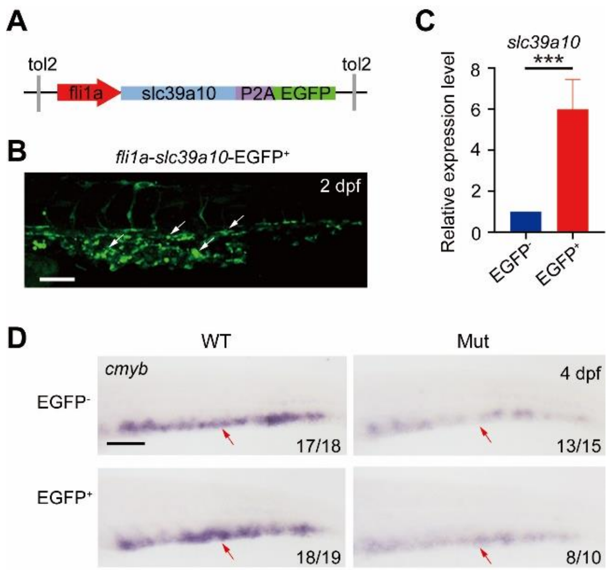

Figure S2. EC-specific overexpression of *slc39a10* failed to reverse the number of HSPCs in *slc39a10* mutant. A, Schematic illustration of the construct of full-length *slc39a10* driven by the *fli1a* promoter. **B,** Confocal imaging showing the expression of Slc39a10 protein indicated by EGFP fluorescence in *fli1a*$^{+}$ ECs in the CHT region at 2 dpf. The white arrows indicated *fli1a*$^{+}$ EC in the CHT. Scale bar, 50 µm. **C,** qPCR showing the mRNA level of *slc39a10* in EGFP^{-} and EGFP^{+} embryos at 4 dpf (n=3 per group). The results are presented as mean ± SD. Student's *t*-test, ***p< 0.001. **D,** WISH showing that the decreased expression of *cmyb* in *slc39a10* mutant embryos could not be rescued by overexpression of *slc39a10* in *fli1a*$^{+}$ ECs. The red arrows indicate *cmyb*$^{+}$ HSPCs in the CHT. Scale bar, 50 µm.

Figure S3

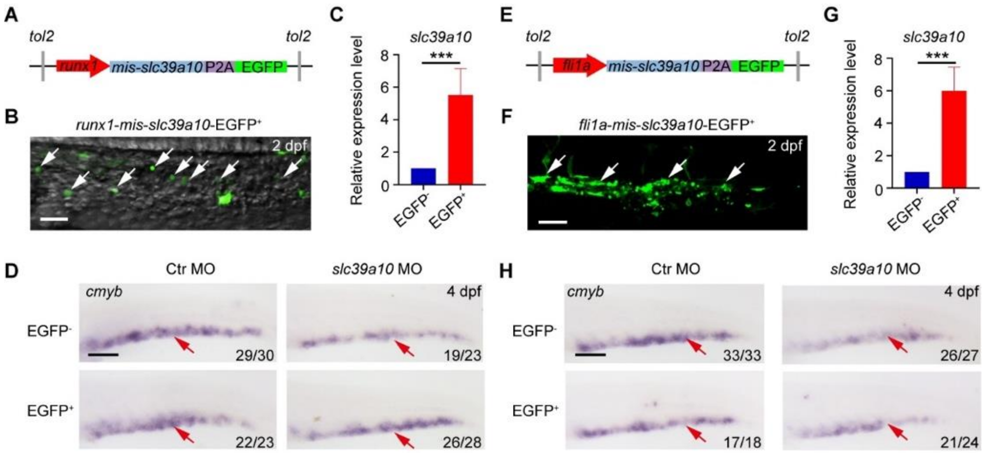

Figure S3. HSPC-specific roles of slc39a10 during HSPC development. A, Schematic illustration of the construct of full-length mismatch-*slc39a10* (*mis-slc39a10*) driven by the *runx1* enhancer. **B,** Confocal imaging showing the expression of *mis*-Slc39a10 protein indicated by the EGFP in *runx1*$^+$ HSPCs in the CHT region at 2 dpf. The white arrows indicated *runx1*$^+$ HSPC in the CHT. Scale bar, 50 μm. **C,** qPCR showing the mRNA level of *slc39a10* in EGFP$^-$ and EGFP$^+$ embryos at 4 dpf (n=3 per group). The results are presented as mean ± SD. Student's *t*-test, ***$p < 0.001$. **D,** WISH showing that decreased expression of *cmyb* in *slc39a10* morphants was partially rescued by overexpression of *mis-slc39a10* in *runx1*$^+$HSPCs. The red arrows indicate *cmyb*$^+$ HSPCs in the CHT. Scale bar, 50 μm. **E,** Schematic illustration of the construct of full-length *mis-slc39a10* driven by the *fli1a* enhancer. **F,** Confocal imaging showing the expression of *mis*-Slc39a10 protein indicated by the EGFP in *fli1a*$^+$ ECs in the CHT region at 2 dpf. The white arrows indicated *fli1a*$^+$ EC in the CHT. Scale bar, 50 μm. **G,** qPCR showing the mRNA level of *slc39a10* in EGFP$^-$ and EGFP$^+$ embryos at 4 dpf (n=3 per group). The results are presented as mean ± SD. Student's *t*-test, ***$p < 0.001$. **H,** WISH showing that decreased expression of *cmyb* in *slc39a10* morphants could not be rescued by overexpression of *mis-slc39a10* in *fli1a*$^+$ECs. The red arrows indicate *cmyb*$^+$ HSPCs in the CHT. Scale bar, 50 μm.

Figure S4

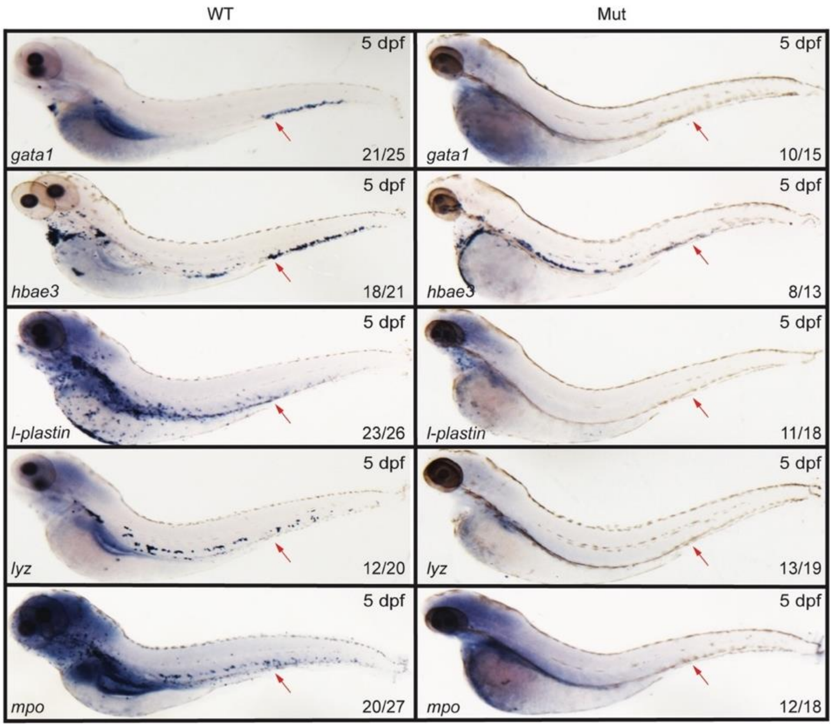

Figure S4. *slc39a10* mutant zebrafish have hematopoietic defects. Whole-mount *in situ* hybridization of *gata1*, *hbae3*, *l-plastin*, *lyz*, and *mpo* mRNA in WT and *slc39a10* mutant sibling embryos measured at 5dpf.

Figure S5

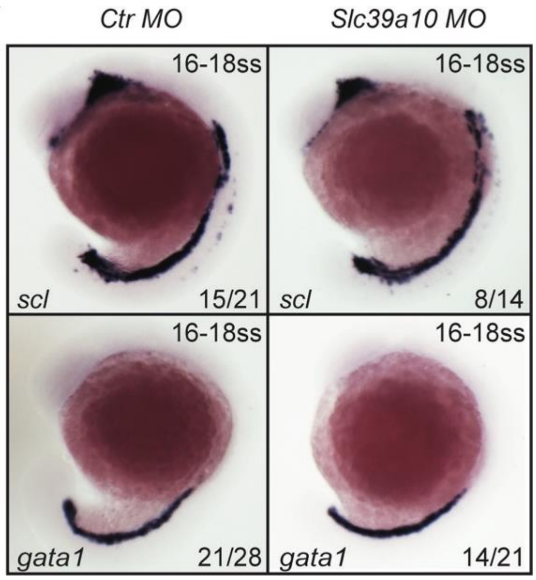

Figure S5. Primitive hematopoiesis is not affected by a loss of *slc39a10*. Whole-mount *in situ* hybridization of *scl* and *gata1* mRNA in 16-18 somite stage embryos injected with a control MO or *slc39a10* MO.

Figure S6

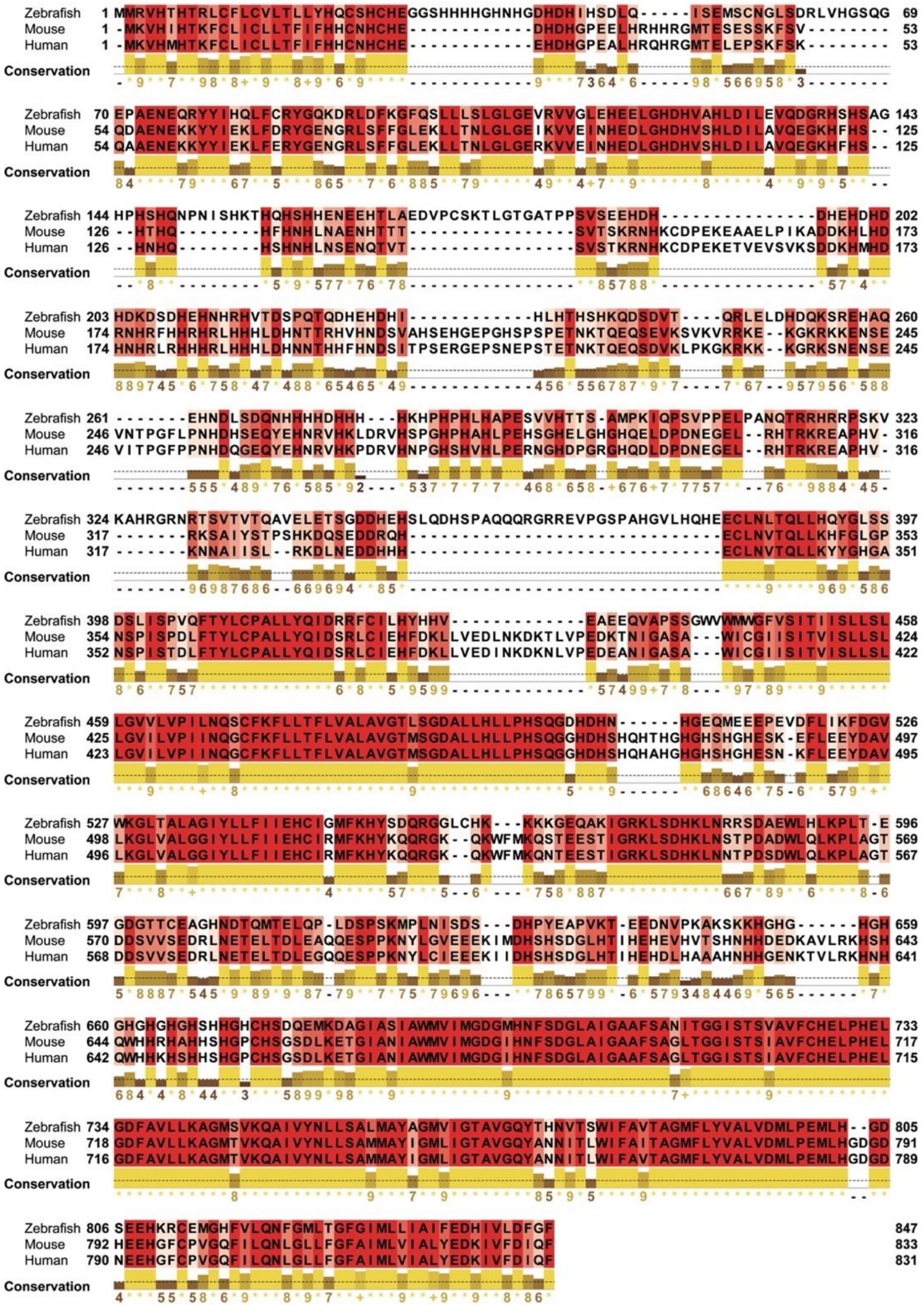

Figure S6. Alignment of amino acid sequences of zebrafish (*Danio rerio*) slc39a10, mouse (*Mus musculus*) Slc39a10, and human (*Homo sapiens*) SLC39A10. Jalview was used to visualize the amino acid sequences, and the MAFFT (Multiple Alignment using Fast Fourier Transform) algorithm was used for the alignment. Shaded regions indicate highly conserved residues in the aligned sequences.

Figure S7

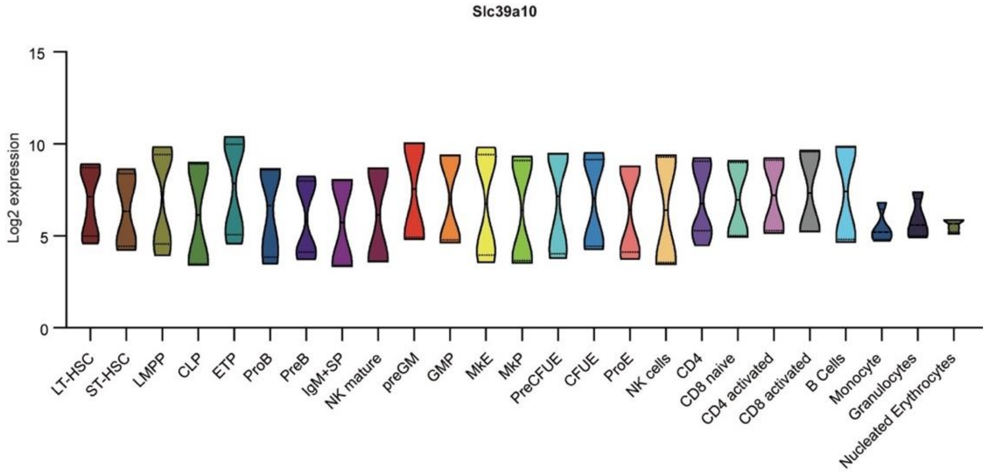

Figure S7. *Slc39a10* expression in the indicated hematopoietic cells types based on publicly available databases. *Slc39a10* mRNA expression data in the indicated cell types were retrieved from BloodSpot (GSE14833 and GSE6506).

Figure S8

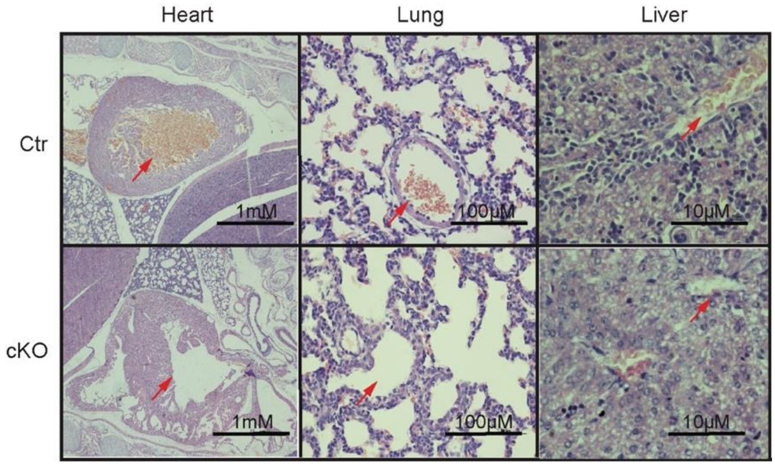

Figure S8. Hematopoietic-specific *Slc39a10*-knockout (cKO) mice have severe anemia in several organs. Heart, lung, and liver sections obtained from 1-day-old control and cKO mouse pups and stained with H&E. The arrows indicate the presence of erythrocytes in the control tissues but absent in the cKO tissues.

Figure S9

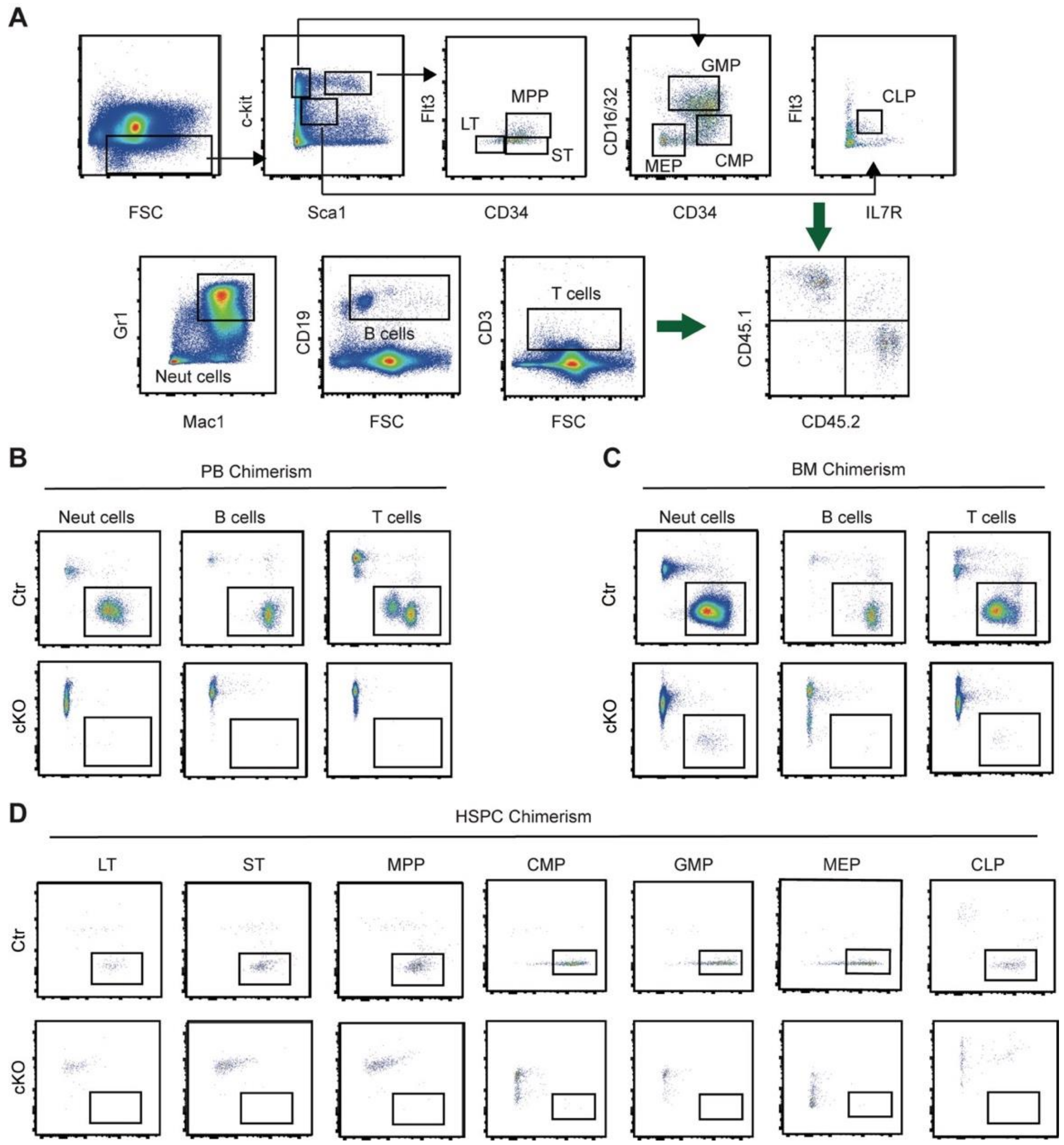

Figure S9. Loss of *Slc39a10* in hematopoietic cells results in an impaired capacity for reconstitution. A, Gating strategy used for the indicated cell lineages in the recipient mice. **B-D**, Representative FACS plots of donor-derived cells in the peripheral blood (**B**) and bone marrow (**C-D**) of recipient mice 16 weeks after co-transplantation with control or cKO HSCs.

Figure S10

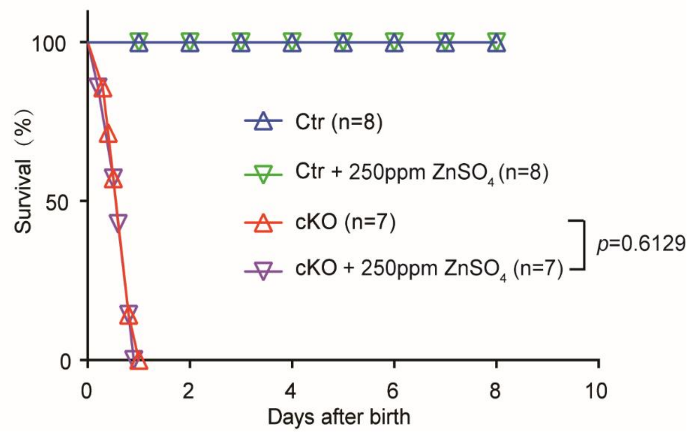

Figure S10. Adding zinc to the drinking water available to pregnant females fails to prolong the lifespan of their cKO pups. Pregnant females were provided with drinking water containing 250 ppm zinc starting on their first day of pregnancy, and the survival of control and cKO pups was plotted for the indicated days following birth. P values of survival were determined using Log-rank test.

Figure S11

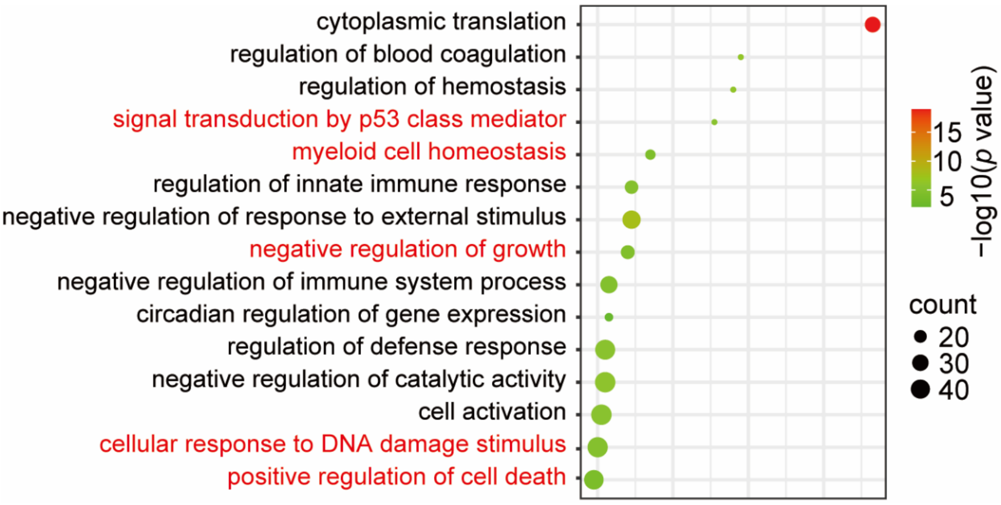

Figure S11. Gene Ontology (GO) analysis of RNA-seq result. RNA-seq data showing the top 15 enriched pathways in fetal LT-HSCs between control and cKO mice (defined as log2 fold change >1 or <-1 and $P<0.05$). The pathways involved in hematopoiesis and cell death are written in red.

Figure S12

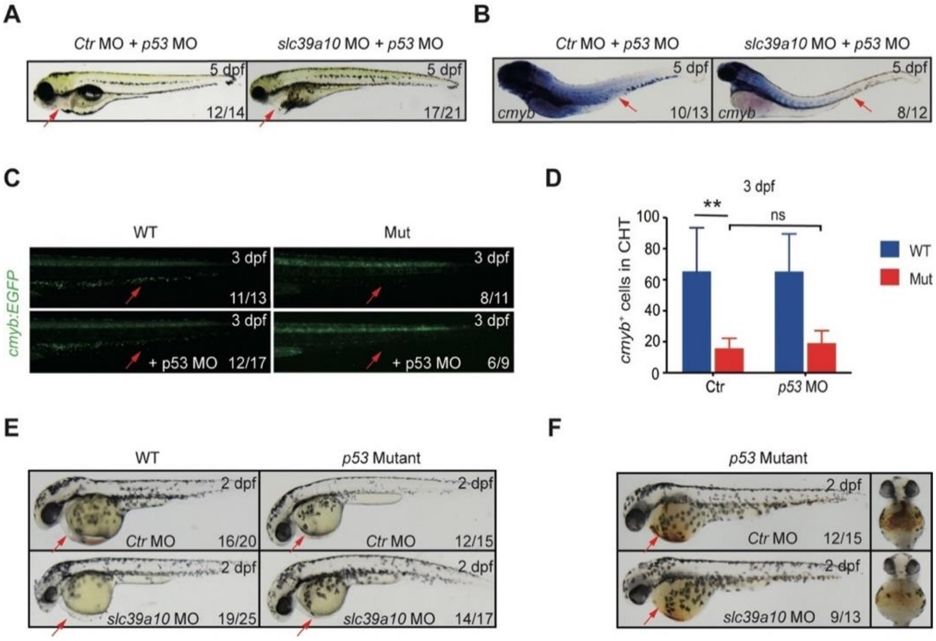

Figure S12. Knocking down *p53* does not restore hematopoiesis in zebrafish. A, Representative images of 5dpf embryos injected with the control MO and *p53* MO (left) or the *slc39a10* MO and *p53* MO (right). The arrows indicate the heart region. **B**, WISH of *cmyb* mRNA in 5dpf embryos injected with the control MO and *p53* MO (left) or the *slc39a10* MO and *p53* MO (right). The arrows indicate the CHT region. **C-D**, Representative images of the CHT region in 3dpf Tg(*cmyb:*eGFP*)* WT and *slc39a10* mutant sibling embryos that were either uninjected or injected with the *p53* MO (**C**), and quantification of *cmyb*$^{+}$ cells in the CHT region at 3dpf (**D**; n= 11 and 12 for wildtype sibling injected control or *p53* MO, n=6 and 6 for *slc39a10* mutant injected control or *p53* MO). **E**, Representative images of 2dpf WT and *p53* mutant sibling embryos injected with the control MO or the *p53* MO. The arrows indicate the heart region. **F**, Representative images of o-dianisidine–stained 2dpf *p53* mutant injected with the control MO or the *p53* MO. The arrows indicate the heart region, and the images at the right show the ventral view. Data in this figure are represent as mean ± SD. The data in **D** were analyzed using a 2-tailed, unpaired Student’s t-test, ***P*<0.01 and ns, not significant (2-tailed, unpaired Student’s t-test).

Figure S13

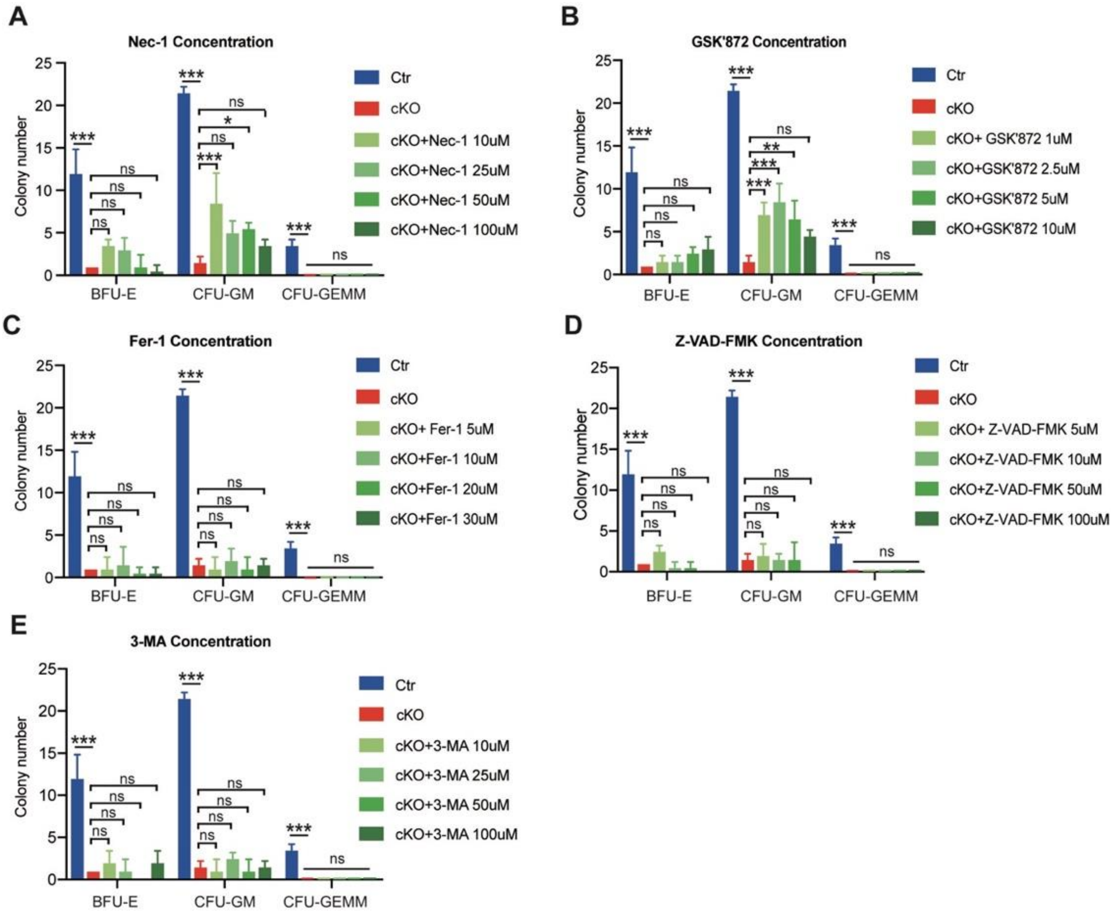

Figure S13. Inhibiting necroptosis partially restores the colony-forming capacity of *Slc39a10*-deficient HSCs. Shown are the number of CFUs formed by FL-HSCs obtained from either control cKO embryos and treated with the indicated concentrations of the necroptosis inhibitor necrostatin-1 (Nec-1; **A**), the necroptosis inhibitor GSK’872 (**B**), the ferroptosis inhibitor ferrostain-1 (Fer-1; **C**), the apoptosis inhibitor Z-VAD-FMK (**D**), or the autophagy inhibitor 3-MA (**E**). N=3 per group for each assay. Data were analyzed using a one-way ANOVA with Tukey’s post hoc test (for multi-group comparisons). **P*<0.05; ***P*<0.01; ****P*<0.001, and ns, not significant.

Supplementary Tables

Table S1. Summary of embryonic lethality in global *Slc39a10*-knockout mice

	**Wildtype**	**Heterozygous**	**Homozygous**	**Total**
E 9.5	13	29	0	42
E 10.5	7	11	0	18
E 12.5	11	25	0	36
E 14.5	4	7	0	11

Heterozygous *Slc39a10* knockout mice were crossed and the offspring embryos were genotyped at different embryonic stages.

Table S2. Sequences of the primers used for zebrafish genotyping

**Gene**	**Forward primer (5' → 3')**	**Reverse primer (5' → 3')**
slc39a6	TGAGTAAAGGATGAAGCGGACAAT	GTACAGTATCGCGGGGTCAATTTC
slc39a10	TGTTCCAGCTGAGAAACACTCTGG	GTGTTGGTGGGTCTTGTGTGAGAT
p53 WT	GATAGCCTAGTGCGAGCACACTCTT	AGCTGCATGGGGGGGAT
p53 Mut	GATAGCCTAGTGCGAGCACACTCTT	AGCTGCATGGGGGGGAA

Table S3. Sequences of the primers used for the zebrafish WISH probes

Gene	Forward primer (5’ → 3’)	Reverse primer (5’ → 3’)
slc39a10	CACCATCATCATGACCACCATCAC	GAGGAGGTAGATTCCTGCTAGTGCAG
hbae3	CCTAAGCCCCAACTCTC	CTCCCTTCAGGTCATCC
gata1	ATGGAGAACTCCTCTGAGCCTTCTC	GCCGTTCATCTTATGGTACAGTCCAC
scl	CTCCCTTCAGGTCATCC	CCGCTGGGCATTTCCGTC
cmyb	ATGGCGAGGCGGCACAGAC	CTGGATAGCAGCAGTGCCATGTTG
runx1	ATGAGCGAGGGTTTGCCTCTG	GTCAGCTCTGGACAGTGTAAAGCG
lyz	CAATATGAGGCTGGCAGTGGTG	TGCATTAGCGCATAATGACTGTAGG
l-plastin	GCTGACGCCCTTCACCATAC	TTCATGTTGCTGCCCAGTTTA
mpo	TCGGGAACAGATCAATACTCTAA	GGCTCAGCAACACCTCCTAAC
rag1	ATCTCCAGACGATTCCGTTATG	CTTAGTTGCTTGTCCAGGGTT

Table S4. Sequences of the primers used for zebrafish RT-qPCR

Gene	Forward primer (5’ → 3’)	Reverse primer (5’ → 3’)
ef1a	TGCATTAGCATAATGACTGTAGG	ATCAAGAAGAGTAGTACCGCTAGCATTAC
mt2	CGTCTAACAAAGGCTAAAGAGGGA	GCAGCAGTACAAATCAGTGCATC
slc39a1	CTATTGGCTTGCAGACGACGA	CCAAGAGGAGACATTATGGCGA
slc39a3	GCCCAAATATCTGACGGACATG	GCTCAATCAGCGTCTGCTTCTC
slc39a4	CCTTCATCGGCCTGTACATC	ATCAGCGTAGGAAGCATGTC
slc39a5	CTCTGGTTGCTCTGGGAATG	AAGATGGCATGAGGCATGAG
slc39a6	GTCATCATGGGAGACGGACT	GGCAAAATCACCGAGTTCAT
slc39a7	GCACTGGAACCTCATTCTCA	AGAAATGCAACAATGCCACC
slc39a8	TTGTCAGTGTCAGCAATCTGGAG	AAAGCAGCCAAGTTAATCACCG
slc39a9	TGCGGAATGTGACGAGCCTTCGC	ACATGTATCCTCGGAGATCGCGTG
slc39a10	TCACCTGCACATGGTGTTCT	ACATCCAAACCCATCCTGAA
slc39a11	GCGGATTTTGGATGGCAGTC	AGCCAGCAGAGACCAATACG
slc39a13	CTTGAAAACTGAGGCTGGGT	CAGGACTGGAAGTGTAAGCC
slc39a14	CGCTGGCTGATATGTTTCCA	TCCTCTGCTATCCAAGCTGT

Table S5. Sequences of the PCR primers used for mouse genotyping

Gene	Forward primer (5’ → 3’)	Reverse primer (5’ → 3’)
Slc39a10$^{fl/fl}$	TGTATCAGATCGACAGCAGACTT	CATGAAGCCAGAACCTGCTA
Vav-Cre	AGATGCCAGGACATCAGGAACCTG	ATCAGCCACACCAGACACAGAGATC
P53$^{fl/fl}$	CACAAAAACAGGTTAAACCCAG	AGCACATAGGAGGCAGAGAC
P21 WT	AAGCCTTGATTCTGATATGGGC	TGACGAAGTCAAAGTTCCACCG
P21 Mut	AAGCCTTGATTCTGATATGGGC	GCTATCAGGACATAGCGTTGGC
P16 WT	GGCAAATAGCGCCACCTAT	GACTCCATGCTGCTCCAGAT
P16 Mut	GGCAAATAGCGCCACCTAT	GCCGCTGGACCTAATAACTTC
Fadd	GAAGCGCATGCGTCTGATT	GCGCTGCAGTAGATCGTGT
Mlkl	CAGCACAAATCCCATCCACTC	TAAACCTGAAGCAGCAGCAAC
Ripk3 WT	ACATGCATGGTCATGCACACACAT	TTGAGACAGGGTCTCTTTTTGGAG
Ripk3 Mut	ACATGCATGGTCATGCACACACAT	GTCGAGGGACCTAATAACTTCGTA

Table S6. Sequences of the primers used for mouse RT-qPCR

Gene	Forward primer (5’ → 3’)	Reverse primer (5’ → 3’)
Hprt	TTTCCCTGGTTAAGCAGTA	TGGCCTGTACCAACACTTCGAGA
Slc39a10	ACTCTGGTTCCTGAAGATAAGAC	GCAGACTAATGACGGTGATAGA
Mt1	GCGTCACCACGACTTCAAC	GTCACATCAGGCACAGCAC
Mt2	GCCTGCAAATGCAAACAATGC	AGCTGCACTTGTCGGAAGC
p21	TGTATATACGCTGCCTGCCC	GGGCCCTACCGTCCTACTAA

Table S7. List of the reagents used in this study

Reagent	Company	Cat Number
mMESSAGE mMACHINE^{TM} SP6	Invitrogen	AM1340
In Situ Cell Death Detection kit	Roche	12156792910
Annexin V Kit	MultiSciences	AP101
$ZnSO_4 \cdot 7H_2O$	Sigma	Z0251-100G
Triton X-100	Sigma	T9284-100ML
TPEN	Sigma	P4413-100MG
Fluo-3, AM, cell permeant	Thermo Fisher Scientific	F24195
MethoCult	STEMCELL Technologies	M3434
StemSpan SFEM Medium	STEMCELL Technologies	09650
Anti-P53	Cell Signaling	9282S
SCF	PeproTech	300-07-250
TPO	PeproTech	300-18-250
FLT3-Ligend	PeproTech	300-19-250
Anti-Rabbit 488	Invitrogen	A-10680
Anti-Kap1	Bethyl Laboratories	A300-767
Anti- Rabbit 555	Invitrogen	A-21428
Anti-Mouse 488	Invitrogen	A-10680
Anit-P21	Cell Signaling	2947T
MitoSox Red	Invitrogen	M36008
anti-phospho-Histone H2A.X	EMD Millipore	05-636-I
anti-pH3-Ser-10	Santa Cruz Biotechnology	sc-8656-R
Anti-GFP	Invitrogen	A-11120
Ferrostatin-1	Selleck	S7243
Necrostatin-1	Selleck	S8037
GSK’872	Selleck	S8465
Z-VAD-FMK	Selleck	S7023
3-Methyladenine (3-MA)	Selleck	S2767

Table S8. Antibody clones and fluorescent labels used for FACS sorting and analysis

Antibody	Fluorescence	Company	Clone
B220 (CD45R)	Biotin	BioLegend	RA3-6B2
CD3e	Biotin	BioLegend	145-2C11
CD5 (Ly-1)	Biotin	BioLegend	53-7.3
CD4	Biotin	BioLegend	GK1.5
CD8a	Biotin	BioLegend	53-6.7
Gr-1 (Ly-6G)	Biotin	BioLegend	RB6-C5
TER-119	Biotin	BioLegend	TER-119
Streptavidin	APC-Cy7	BioLegend	
B220 (CD45R)	AlexaFluor700	BioLegend	RA3-6B2
CD3e	AlexaFluor700	BioLegend	145-2C11
Gr-1 (Ly-6G)	AlexaFluor700	BioLegend	RB6-C5
TER-119	AlexaFluor700	BioLegend	TER-119
CD11b (Mac-1)	AlexaFluor700	BioLegend	M1/70
Sca-1	PE-Cy7	BD Biosciences	D7
CD117 (c-Kit)	APC	BioLegend	2B8
CD135 (Flt3)	PE	BioLegend	A2F10
CD135 (Flt3)	Biotin	BioLegend	A2F10
CD34	PE	BioLegend	RAM34
CD34	FITC	BD Biosciences	RAM34
CD127 (IL-7R)	BV421	BioLegend	A7R34
CD16/32	PerCP-Cy5.5	BioLegend	93
CD71	PerCP-Cy5.5	BD Biosciences	C2
CD71	PE	BioLegend	C2
Ter119	APC	BioLegend	TER-119
CD44	PE-Cy7	BioLegend	IM7
B220	FITC	BioLegend	RA3-6B2
B220	APC	BioLegend	RA3-6B2
CD19	PE-Cy7	BioLegend	1D3

CD11b (Mac-1)	PE	BioLegend	M1/70
CD11b (Mac-1)	APC	BioLegend	M1/70
CD11b (Mac-1)	FITC	BioLegend	M1/70
Gr-1	PE	BioLegend	RB6-8C5
Gr-1	APC	BioLegend	RB6-8C5
CD3e	AlexaFluor700	BioLegend	145-2C11
CD45.2	APC	BioLegend	104
CD45.2	BV650	BioLegend	104
CD45.2	PerCP-Cy5.5	eBioscience	104
CD45.2	Biotin	eBioscience	104
CD45.1	PE	eBioscience	A20
CD45.1	Biotin	eBioscience	A20
